# Updating the critical steps of the quality implementation framework: an umbrella review of reviews

**DOI:** 10.1186/s43058-026-00954-7

**Published:** 2026-05-27

**Authors:** Kathrine Hald, Anne Gulbech Ording, Martin Jorsal, Julie Midtgaard, Louise A. Ellis, Samantha Spanos, Lisa Pagano, Georgia Fisher, Abraham Wandersman, Jeffrey Braithwaite, Søren Paaske Johnsen

**Affiliations:** 1 Danish Center for Health Services Research, Department of Clinical Medicine, Aalborg University, Aalborg, Denmark; 2 Medical Diagnostic Centre, University Research Clinic for Innovative Patient Pathways, Silkeborg, Denmark; 3 Aalborg University Hospital, Aalborg, Denmark; 4 Centre for Applied Research in Mental Health Care (CARMEN), Mental Health Center Glostrup, Copenhagen University Hospital – Amager and Hvidovre Hospital, Hvidovre, Denmark; 5 Danish Concussion Center, Copenhagen, Denmark; 6 Department of Clinical Medicine, University of Copenhagen, Copenhagen, Denmark; 7 Centre for Healthcare Resilience and Implementation Science, Australian Institute of Health Innovation, Macquarie University, Sydney, New South Wales Australia; 8 Wandersman Center, Columbia, South Carolina, USA; 9 University of South Carolina, Columbia, South Carolina, USA

**Keywords:** Implementation science, Quality implementation framework, Process model, Umbrella review of reviews

## Abstract

**Background:**

The Quality Implementation Framework (QIF) is a widely used process model in implementation science (IS). Since its publication in 2012, the field of IS has expanded considerably, yet QIF has never undergone formal revision. Given recent advances and the complexity in implementation research and practice, this study examines whether QIF continues to capture the full scope of implementation quality and its challenges.

**Methods:**

An umbrella review of reviews was conducted on literature published between 2012 and 2025. Eligible articles reported on the development, application, or update of implementation frameworks within healthcare or social science. Data were deductively mapped to the four phases and 14 steps of the original QIF and inductively mapped to identify knowledge not included in the original framework.

**Results:**

A total of 15 reviews met the inclusion criteria. Most aligned with the core structure of QIF, supporting its continued relevance. However, several reviews highlighted the need to add a pre-implementation phase focusing on evidence appraisal, and a post-implementation phase addressing sustainability. Four cross-cutting domains (service user, intervention deliverer, context, and technology) were identified as critical factors throughout the implementation process.

**Conclusions:**

An updated version of QIF is proposed, building on the original framework while introducing two new phases and four cross-cutting domains. This expanded model reflects recent developments in the literature and provides more comprehensive guidance to support implementation across complex real-world settings; it has important implications for implementation research and implementation practice.

**Registration:**

PROSPERO registration number: CRD42023475994.

**Supplementary information:**

The online version contains supplementary material available at 10.1186/s43058-026-00954-7.

**Table:** 

Contributions to the literature	
• This umbrella review of reviews offers an updated version of the Quality Implementation Framework (QIF), a process model widely used in implementation science.	
• Emphasizes the continued relevance of process models in guiding real-world implementation, particularly for translating theory into practice.	
• Introduces two additional phases to better reflect current implementation challenges. One focusing on assessing evidence before implementation, and one addressing long-term sustainability.	
• Enhances the applicability of QIF by embedding four cross-cutting domains (service user, intervention deliverer, context, and technology) as continuous considerations across all phases and steps of implementation.	

## Background

The field of implementation science is defined as *“a science aiming to apply concepts and measures […] to the process of introducing newly developed clinical evidence and innovation into practice”* [1]. Building on this definition, implementation science (IS) aims to operationalize the uptake of evidence-based practice (EBP) through structured methods and a nuanced understanding of context [1–3]. The field applies to several sectors, however it is particularly useful in healthcare and social science, where the implementation of research evidence occurs in complex adaptive systems [2, 4]. Although IS has existed as a discipline for several decades [5–9], a substantial knowledge-to-action gap persists in healthcare [10]. Consequently, there is an increasing need for implementation approaches that align more closely with the complex realities of implementing EBP and that more explicitly address practical aspects of implementation, e.g., the *how to* [11].

**Fig. 1:**
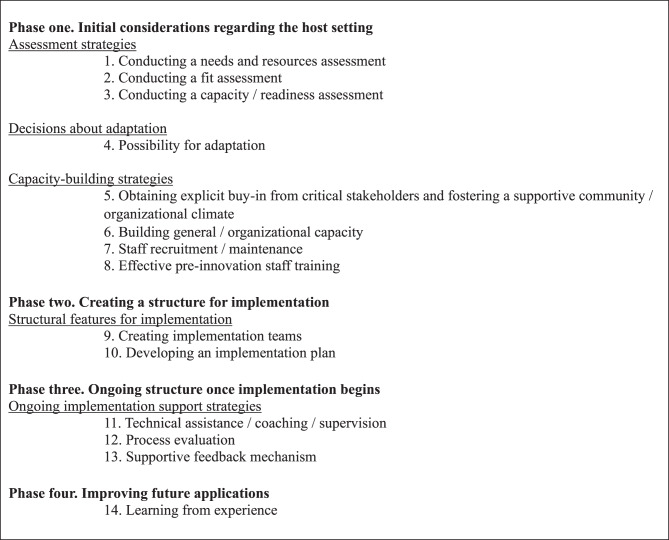
The quality implementation Framework (QIF) as developed and published by Meyers et al. In 2012 [15]

IS is not lacking in theoretical approaches; rather, it is characterized by an abundance of theories, models, and frameworks (TMFs) that vary in scope and purpose, but all intended to support the understanding and organisation of implementation efforts. There are five categories of TMFs: Process models, which focus on the *how to* of implementation; Determinant frameworks, focusing on factors affecting implementation outcomes; Classic theories, focusing on change mechanisms; Implementation theories, focusing on theoretical aspects of implementation; and Evaluation frameworks, focusing on structuring implementation evaluation [3]. A substantial number of influential TMFs have been developed during the last decades. Several of these have been widely adopted in implementation research and practice [8, 9, 12–14]. However, most do not thoroughly address the natural progression of the implementation process. That is, they are descriptive and analytical, but they lack a practical approach (i.e., a clear roadmap) on *how to* when implementing [3, 15]. The Quality Implementation Framework (QIF), published in 2012, was based upon a synthesis of 25 existing TMFs. It was developed to identify the critical steps associated with high-quality implementation and to provide structured guidance for carrying out implementation processes. [15]. It can be classified as a process model [3, 15] and can be applied as a tool for guiding implementation efforts in practice [15, 16]. The QIF has been applied across a range of sectors and settings and has proven useful both for planning and evaluating the implementation process [16]. QIF consists of four phases and 14 steps. Phase one relates to the initial considerations and contains eight steps. Phase two focuses on creating a structure and contains two steps. Phase three deals with the ongoing implementation and contains three steps. Phase four explores how to learn from experience and contains one step [15]. Phase one and phase two occur prior to active implementation, with Phase three and four conducted during and after implementation. An overview of the full framework is provided in Fig. 1.

Since the development of QIF, IS has progressed considerably, both conceptually and empirically. As a result, numerous frameworks have been revised or updated from their original form to reflect advances in the field [17–19]. However, QIF has not undergone a formal update despite being widely cited over the years. A search in the database Web of Science shows that the original article [15] has been cited more than 800 times since it was published in 2012. Moreover, the framework has been widely used in practice, particularly in Scandinavia [20–22], and has served as the foundation for the development of new toolkits [20, 21]. This continued application and adaptation indicate the framework’s relevance and support the need for an updated and strengthened version of the QIF. Building on this, the present study aimed to develop a comprehensive synthesis that not only advances conceptual understanding of implementation processes but also supports their practical application in real-world settings. Given the evolving complexity of implementation evidence, it remains unclear whether QIF still captures the full scope of implementation challenges. Thus, our specific aims were [23]:

- To conduct an umbrella review of published reviews in the fields of healthcare and social science since 2012 and synthesize their findings according to the four phases and 14 steps of the QIF.
- To summarize new knowledge on the phases and steps of the implementation process and propose recommendations for an updated QIF.
- To guide future research efforts and outline the practical implications of our findings to improve future implementation efforts.

## Methods

### Study design

At the end of 2012, a retrospective free-text search conducted in PubMed in May 2025 using the search term ‘implementation science’ identified 706 results. As of May 2025, this number had risen to 13,622, highlighting the rapid expansion of the IS literature. In response to this substantial growth, we undertook an umbrella review of reviews (RORs) to synthesize and critically examine developments in the field since the publication of QIF.

Although umbrella RORs and overviews of reviews are typically guided by established methodological standards, such as the Cochrane guidance [24] and the Preferred Reporting Items for Overviews of Reviews (PRIOR) statement [25], these are primarily designed for reviews of systematic, quantitative evidence. In the present study, our inclusion criteria encompassed not only systematic reviews but also scoping and narrative reviews, given the conceptual and exploratory nature of our research questions. Consequently, it was neither appropriate nor feasible to apply existing guidelines in their entirety. Instead, we adopted a flexible yet systematic approach, drawing on established guidance where relevant while adapting procedures to ensure methodological rigour and alignment with the aims of our study. The PRIOR checklist was used to guide reporting of the overview of reviews and to ensure transparency and completeness of reporting. The completed PRIOR checklist is available in Additional File 1.

The study was registered a priori on the PROSPERO International Prospective Register of Systematic Reviews (Registration number: CRD42023475994) and has been published as a peer-reviewed protocol article [23].

### Review questions

We sought to examine the following research questions [23]:

- In published reviews within the healthcare and social science literature since 2012, how do implementation frameworks map to the four phases and 14 steps of the original QIF?
- Do the above frameworks outline any phases or steps that are additional to those outlined in the original QIF?
- Can the literature published since 2012 offer new knowledge and suggestions on the *how to* of implementation, which could guide future implementation efforts?

### Eligibility criteria

We included peer-reviewed systematic reviews, scoping reviews, and narrative reviews that reported on the development, application, or update of implementation frameworks within healthcare or social science settings. Articles were eligible if they focused on implementation strategies targeting human populations and addressed the *how to* of implementation such as procedural steps, guiding principles, or contextual considerations relevant to the implementation process [23].

Implementation frameworks were defined as structured approaches, either empirically derived or theoretically grounded, that describe key stages or strategies for translating research evidence into practice. Articles that examined only intervention fidelity, clinical outcomes, or the delivery of interventions without a specific focus on implementation frameworks were excluded. As were editorials, protocols, commentaries, conference abstracts, and grey literature [23].

The primary outcome of interest was the content of the included articles related to the implementation frameworks they developed, applied or updated, specifically the steps, phases, and strategies they proposed. This content was structured and mapped against the four phases and 14 steps outlined in QIF [15, 23].

Only articles published in English language from January 2012 up to and including December 2025 were included, as this time frame reflects the period following the original publication of QIF and aims to capture subsequent developments in IS since then [15, 23].

### Information sources and literature search

A systematic literature search was conducted across four electronic databases: PubMed, Scopus, PsycINFO, and Web of Science. The search strategy was developed in collaboration with an experienced research librarian. It combined MeSH terms and free-text keywords related to implementation AND framework AND review. The full search strategies for all four databases are available in Additional File 2. The initial literature search was carried out in late 2023, and an updated search was completed in January 2025 as well as in January 2026. All search results were imported into the Covidence software [26] for removal of duplicates, screening, and data management [23].

### Literature selection

Using the outlined eligibility criteria, four reviewers (KH, AGO, MJ, and SPJ) independently screened titles and abstracts, followed by full-text screening of the remaining references. Any discrepancies were resolved through discussion. All excluded full-text references were documented with a reason for exclusion, and a complete list of all full-text exclusions is available in Additional File 3. The screening procedure was pilot-tested using 100 randomly selected references at the title/abstract stage and 20 at the full-text stage. The entire screening process was tracked using a flow diagram in accordance with the Preferred Reporting Items for Systematic Reviews and Meta-Analyses (PRISMA) guidelines [23, 27].

### Study quality assessment

The methodological quality was assessed using the Joanna Briggs Institute (JBI) Critical Appraisal Checklist for Systematic Reviews and Research Syntheses [28]. Three reviewers (KH, AGO, and SPJ) independently conducted the quality assessments. Any discrepancies were resolved through discussion. The results of the critical appraisal were used to assess the methodological rigor of the included reviews and to inform the interpretation of the findings; they were not applied as exclusion criteria. The completed checklists for all included articles were compiled and are available in Additional File 4.

### Data extraction

Data were extracted using a structured extraction sheet developed a priori and pilot-tested on 10% of the included articles. Three reviewers (KH, AGO, and SPJ) independently extracted data from all included articles. Following the independent extraction, the reviewers met to compare their extracted data and finalise a consensus-based extraction. Any discrepancies were resolved through discussion.

The following data were extracted, where available:

1. Background information: Title, first author, institution, country, publication year, journal, aim and research questions.
2. Review characteristics: Type of review, population, inclusion and exclusion criteria and implementation framework(s) included.
3. Mapping of content from the included articles to the four phases and 14 steps of QIF.
4. Mapping of content additional to QIF from the included articles.

The procedures and findings related to steps 3 and 4 are described in greater detail in the section ‘Data synthesis and analysis’.

### Data synthesis and analysis

A narrative synthesis was conducted using a *directed content analysis approach* [29]. This method served as a guiding analytical approach, allowing for a primarily deductive coding process structured around QIF [15]. The analysis was grounded in the four phases and 14 steps of QIF which remained the primary analytical structure throughout our study [15, 23]. Data were deductively mapped according to the predefined QIF phases and steps. Data that provided additional knowledge to QIF on the *how to* of implementation were mapped inductively. For the inductive data, reviewers independently coded content that did not align with the four QIF phases and 14 steps and generated preliminary descriptive labels. Codes were subsequently compared and discussed in joint meetings to identify conceptual similarities and differences. In cases of disagreement, the reviewers revisited the original articles together to re-examine the relevant sections before reaching consensus. Where codes overlapped or were phrased differently but reflected similar underlying themes, they were merged through discussion and agreement. No formal codebook was developed a priori for the inductive analysis. Instead, themes were refined iteratively throughout the analytic process. However, the overall synthesis was based on the structure of QIF, and the purpose of the inductive component was not to reinterpret the framework, but to assess its completeness considering recent literature. The synthesis was presented in narrative form and accompanied by a table to demonstrate how the included articles align with QIF. To ensure consistency with the original framework, we deliberately adopted the structure and reporting style of the original QIF article [15], including the design of the summary table showing which QIF phases and steps were present in each article. The purpose of the mapping in the present study was structural comparison in order to update the QIF, rather than to assess the degree or quality of coverage within each article. A step was coded as present (+) when it was explicitly described or substantively addressed in the article. In cases of uncertainty, both reviewers revisited the original articles together and discussed the interpretation until consensus was reached. While interpretive judgement was inherent to the process, the binary approach was considered appropriate given the aim of the study. Based on the results of the synthesis, we proposed an updated model of QIF that reflects new insights into the phases and steps of the implementation process [23].

### Deviations from the protocol

The PRISMA guideline [27] was initially considered to ensure structured and consistent reporting of the study [15]. However, the PRIOR guideline [25] was ultimately applied, as it is designed to support reporting of overviews of reviews and was therefore more appropriate for the study design. The PRISMA guideline [27] was nonetheless used to guide the development of a flow diagram, as previously described.

For the assessment of methodological quality, the AMSTAR 2 assessment instrument’ [30] was originally selected [23]. During the appraisal process, it became evident that AMSTAR 2 [30] was not suitable for this study, as it is primarily intended for evaluating systematic reviews of quantitative intervention studies. Given the inclusion of articles with a more conceptual or qualitative orientation, JBI Critical Appraisal Checklist for Systematic Reviews and Research Syntheses’ [28] was used instead.

Regarding data synthesis, *the Framework Method* [31] was initially proposed [23]. However, *Directed Content Analysis* [29] was found to be more appropriate given the aim of our study. While *the Framework Method* [31] is particularly suited for primary qualitative data, this article involved secondary data extracted from articles mapped to pre-defined phases and steps of QIF [15]. *Directed Content Analysis* [29] supported mapping within the structure of QIF, while still allowing for the inductive identification of new or expanded phases and steps when data fell outside the original framework.

## Results

### Search results

**Fig. 2:**
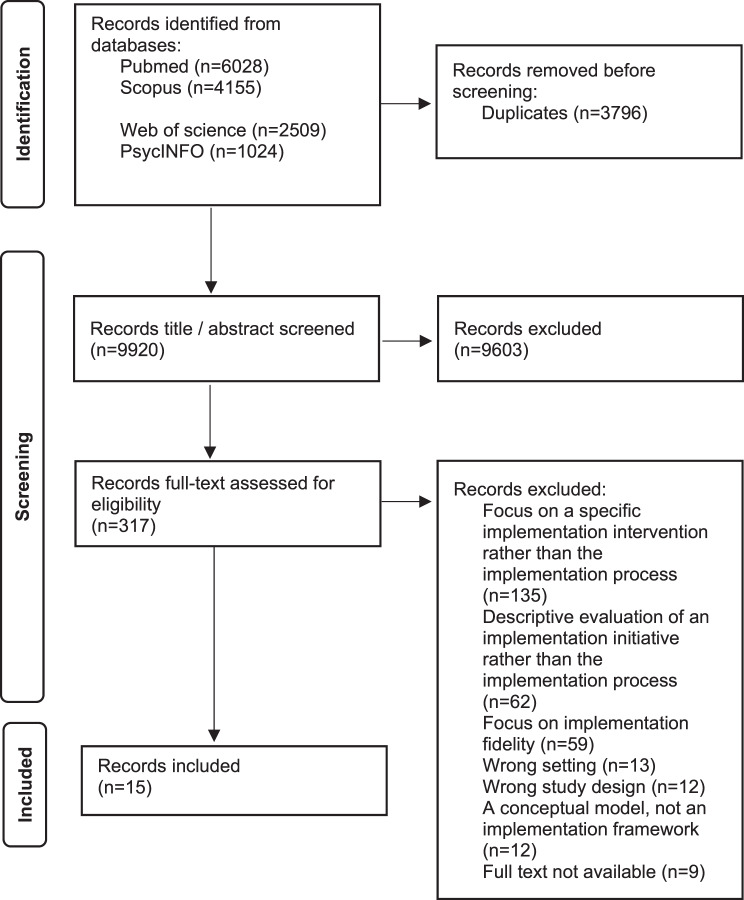
PRISMA flow diagram [27]

After de-duplication, 9,920 records identified through the systematic literature search were screened by title and abstract. Of these, 317 were assessed in full text. A total of 15 records met the eligibility criteria and were included in the study. A list of all full text excluded records with reasons for exclusion, is available in Additional File 3. Figure 2 presents the study selection process, and Table 1 provides an overview of the included articles.

**Table 1:** Overview of included articles

Author, year, country	Aim(s)	Population	Review design	Guiding framework(s)
**Albers** [32], 2017, Australia.	- To identify studies employing an implementation framework in child, youth, and family services. - To map the literature to better understand these frameworks and the ways in which they are being applied. - To ascertain the ways in which implementation frameworks are being tested and to describe the current state of evidence surrounding their use in the field.	Children, adolescents, and their families.	A Scoping review.	The Active Implementation Frameworks (AIF), The Availability, Responsiveness and Continuity Organizational and Community Intervention Model (ARC), The Community Development Team (CDT), The Consolidated Framework for Implementation Research (CFIR), The Exploration Preparation Implementation and Sustainment (EPIS) framework, The Getting to Outcomes (GTO) framework, The Interactive Systems Framework (ISF), The Practical, Robust Implementation and Sustainability Model (PRISM).
**Chaudoir** [33], 2013, USA.	To identify a multi-level framework that captures the predominant factors that impact implementation outcomes, conduct a systematic review of available measures assessing constructs subsumed within these primary factors, and determine the criterion validity of these measures in the search articles.	No specific population stated but the review is focusing on health innovations including provider and patient level measures.	A systematic review.	Not stated/N/A. A framework is developed in the article.
**Escoffery** [34], 2023, USA.	The purpose of this scoping review was to collect definitions of context and identify salient determinants of outer context found in dissemination and implementation theories, models, and frameworks.	Not stated.	A Scoping review.	A Conceptual Model for the Diffusion of Innovations in Service Organizations, A Conceptual Model of Evidence-Based Practice Implementation in Public Service Sectors, A Conceptual Model of Implementation Research, A Conceptual Model of Knowledge Utilization, A Framework for Analyzing Adoption of Complex Health Innovations, A Framework for the Dissemination & Utilization of Research for Healthcare Policy & Practice, A Framework of Dissemination in Health Services Intervention Research, Availability, Responsiveness, & Continuity (ARC) Model, Blueprint for Dissemination, CDC DHAP’s Research-to-Practice Framework, Collaboration Framework for Community-Based Knowledge Translation, Conceptualizing Dissemination Research and Activity: Canadian Heart Health Initiative, Consolidated Framework for Implementation Research (CFIR), Coordinated Implementation Model, Critical Realism & The Arts Research Utilization Model (CRARUM), Davis’ Pathman-PROCEED Model, Diffusion of Innovation, Dissemination of Evidence-Based Interventions to Prevent Obesity, Effective Dissemination Strategies, Framework for Knowledge Translation, Framework for the Dissemination & Utilization of Research for Healthcare Policy & Practice, Framework of Dissemination in Health Services Intervention Research, Health Promotion Research Center Framework, Integrated Knowledge Translation Framework, Knowledge Exchange Framework, Knowledge Integration Process, Knowledge Transfer Framework for AHRQ Patient Safety Portfolio, Knowledge Translation Model of Tehran University of Medical Sciences, Linking Systems Framework, Marketing and Distribution System for Public Health, Model for Locally Based Research Transfer Development, Multi-Level Conceptual Framework of Organizational Innovation Adoption, NCHPAD Knowledge Adaptation, Translation, and Scale-up (N-KATS) Framework, OutPatient Treatment in Ontario Services (OPTIONS) Model, Pathways to Evidence-Informed Policy, Policy Framework for Increasing Diffusion of Evidence-Based Physical Activity Interventions, Practical, Robust Implementation and Sustainability Model (PRISM), Precaution Adoption Process Model, Quality Improvement Framework, RAND Model of Persuasive Communication and Diffusion of Medical Innovation, Real-World Dissemination Strategies, Research Development Dissemination and Utilization Framework, Social Cognitive Theory, Social Ecology Model for Health Promotion, Streams of Policy Process, The Precede-Proceed Model.
**Huybrechts** [35], 2021, Belgium.	To map the similarities and differences of various frameworks and models, in order to find key constructs that form the foundation of an implementation framework or model that is to be developed.	It does not define a traditional population as it is a narrative review of theoretical models and frameworks. However, the setting is primary care.	A narrative review.	Medical Research Council Guidance Conceptual Model of Evidence-Based Practice Implementation in Public Service Sectors, Consolidated Framework for Implementation Research (CFIR), NCCDPHP Knowledge to Action Framework for Public Health, Research Utilization Model (modified from Rogers), Active Implementation Frameworks, Stetler Model of Research Utilization Generic Implementation Framework (GIF), ACE Star Model of Knowledge Transformation, Model for Large Scale Knowledge Translation, Advancing Understanding of Mechanism of Change in Implementation Science Organizational Model for Transformational Change in Health Care Systems, The Ottawa Model of Health Care Research, Quality Implementation Framework (QIF), IOWA Model.
**Li** [36], 2018, Canada.	To identify the most commonly reported organizational contextual features that influence the implementation of evidence-based practices across healthcare settings, and to describe how these features affect implementation.	People working across healthcare settings.	A systematic, integrative review.	Technology Acceptance Model, PARiHS framework (Promoting Action on Research Implementation in Health Services), Organizational Learning Theory, Rogers’ Diffusion of Innovations Theory, Organizational Framework of Innovation Implementation, CFIR (Consolidated Framework for Implementation Research), Weiner’s Conceptual Model of Organizational Readiness for Change, Absorptive Capacity Framework, Grol and Wensing’s Model of Implementation, Greenhalgh’s Multilevel Model of Diffusion of Innovations, Prochaska, Velicer’s Transtheoretical Model of Change.
**Mahmoud** [37], 2023, USA.	This paper presents OPTIC as a framework to guide the conceptualization and implementation of telebehavioral health (TBH) in a comprehensive, structured, and accessible manner.	Individuals in need of telebehavioral health interventions.	Not stated but the aim suggests that it is a narrative review. The review summarizes various frameworks and develop the OPTIC framework. It is stated that the development not only rely on the literature but also on the authors’ long experience. The review does not follow any pre-defined structures such as the structures of a systematic or Scoping review.	Telehealth evaluation framework, Conceptual framework for telemedicine satisfaction, TBH competency framework for TBH professionals, Framework that incorporates barriers and facilitators to telehealth utilizations, across six medical specialties, Framework for evaluating telehealth system implementation, Summary of approaches and outcome measures for evaluating TBH programs, Systematic approach to effectively implement telemedicine in a large multicenter integrated healthcare system, An emergent research and policy framework for telehealth.
**McHugh** [38], 2020, Irland.	To identify measures of outer setting and its CFIR-delineated constructs used in behavioral and mental health research and assess the psychometric properties of those measures.	No population per se but the setting was mental health services, psychiatry and substance abuse.	A systematic review.	The Consolidated Framework for Implementation Research (CFIR).
**Nair** [39], 2025, Sweden.	This study aims to explore the activities typical to implementation of AI-based systems to develop an AI implementation process framework intended to guide healthcare professionals.	No specific population was included. The focus of the study is on AI in healthcare.	A literature review.	The Quality Implementation Framework (QIF).
**Nilsen** [40], 2019, Sverige.	The aim of this scoping review was to identify and examine determinant frameworks used in implementation science to address four issues: 1) How were the frameworks developed? 2) What terms do they use to denote contextual determinants for implementation? 3) How is the context conceptualized? 4) Which context dimensions are applied across the frameworks?	No specific population was included. The focus of the study is on context.	A Scoping review.	PARIHS/i-PARIHS, Cabana et al., Mäkelä & Thorsen, Grol & Wensing, 2004, Fleuren et al., Greenhalgh et al., TDF (Theoretical Domains Framework), Wensing et al., AIF (Active Implementation Frameworks), NICS (National Institute of Clinical Studies), Cochrane et al., Nutley et al., PRISM (Practical, Robust Implementation and Sustainability Model), CFIR (Consolidated Framework for Implementation Research), Gurses et al., 2010, SURE (Supporting the Use of Research Evidence), TICD (Tailored Implementation for Chronic Diseases).
**Ostermeier** [41], 2023, Canada.	The aim of this scoping review was to explore the implementation models and frameworks used to develop, explore, and/or evaluate community-based physical activity programs for children ages 5 to 12 years. The primary objective of this study was to identify the models and frameworks employed by researchers and program developers to support the implementation of community-based physical activity programs for children. As a secondary objective, the key components of the models and frameworks, and how the models and frameworks have been used in practice are highlighted.	Children aged 5 to 12 years.	A Scoping review.	Analysis Grid for Environments Linked to Obesity (ANGELO) Framework, Behaviour Change Wheel, Community-Based Prevention Marketing (CBPM) Framework, Foster-Fishman et al. Systems Framework, Integrated Capacity Building Framework, Knowledge-to-Action (KTA) Framework, Life Needs Model, Management Model for Sport and Physical Activity Community-Based Partnerships, Social Marketing Model, Strategies To Enhance Practice – Physical Activity (STEPs-PA), Typology of Cultural Adaptation and Programme Theory of Adapted Health Promotion Interventions, A+ Quality Improvement Toolkit, RE-AIM Framework, Hybrid Type 3 Evaluation Design.
**Piat** [42], 2021, Canada.	No known systematic review, to date, has been published on how recovery has been implemented into services from an implementation science perspective. To address this knowledge gap, it was deemed appropriate to employ a systematic mixed studies review to ensure that we captured the breadth of evidence across research designs.	Adults with serious mental illness.	A systematic mixed studies review.	The Consolidated Framework for Implementation Research (CFIR).
**Richter** [43], 2022, Sweden.	This scoping review aimed at synthesizing the literature on the implementation of school-based mental health services (SBHMS). By doing so, we aim to increase the understanding of the systemic conditions and factors that affect the implementation of SBHMs.	Children and adolescents suffering from mental illness.	A Scoping review.	The Consolidated Framework for Implementation Research (CFIR).
**Shierk** [44], 2025, USA.	This scoping review aimed to define goal-directed training (GDT) and its impact on outcomes for children with cerebral palsy (CP), and to develop a structured framework outlining its core components for effective implementation.	Children with cerebral palsy.	A Scoping review.	The International Classification of Functioning, Disability and Health (ICF) Framework.
**Toms** [45], 2019, United Kingdom.	Using existing implementation frameworks, this critical literature review scoped the DBT implementation literature to develop and refine a bespoke DBT implementation framework.	Stakeholders involved with dialectical behavior therapy (therapists, patients, health service units etc.).	A literature review.	PARIHS – Promoting Action on Research Implementation in Health Services, CFIR – Consolidated Framework for Implementation Research, Core Implementation Components model, Dialectical behaviour therapy-specific guidance.
**Varsi** [46], 2019, Norway.	To summarise evidence from empirical studies regarding: - Which implementation strategies are used when implementing eHealth interventions for patients with chronic illnesses living at home - Implementation outcomes - The relationship between implementation strategies, implementation outcomes, and degree of implementation success.	Patients with chronic illnesses living at home and participating in eHealth programs.	A realist systematic review.	Reach, Effectiveness, Adoption, Implementation, Maintenance (RE-AIM) Framework, Normalization Process Theory (NPT), Consolidated Framework for Implementation Research (CFIR), Structuration Theory, Promoting Action on Research Implementation in Health Services (PARIHS) Framework, Plan-Do-Study-Act (PDSA) Cycle, Theoretical Domains Framework (TDF), Technology Acceptance Model (TAM).

### Literature characteristics

The 15 included articles [32–46] were published between 2013 and 2025. The country of origin of the first author’s affiliated institution ranged from the United States (*n* = 4, 26.7%), Canada (*n* = 3, 20.0%), Sweden (*n* = 3, 20.0%), Australia (*n* = 1, 6.7%), Belgium (*n* = 1, 6.7%), Ireland (*n* = 1, 6.7%), Norway (*n* = 1, 6.7%), and the United Kingdom (*n* = 1, 6.7%). The review types comprised a mix of scoping reviews (*n* = 6, 40.0%), narrative reviews (*n* = 2, 13.3%), systematic reviews (*n* = 2, 13.3%), a literature review (*n* = 2, 13.3%), a realist systematic review (*n* = 1, 6.7%), a systematic integrative review (*n* = 1, 6.7%), and a systematic mixed studies review (*n* = 1, 6.7%). Due to rounding, percentages do not add up to exactly 100%. The populations studied varied across the fields of healthcare and social science. In some cases, no specific population was identified; instead, the focus was on the setting or context. Several TMFs informed the work conducted in the included articles. A more detailed description of the articles, including their aims, is available in Table 1.

The overall quality of the articles included was high. Two articles [34, 37], however, received a notably lower quality appraisal rating. Nonetheless, they were retained, as the reviewers (KH and AGO) assessed that they offered highly relevant perspectives on key aspects of the implementation process [34] and presented a valuable and applicable framework [37]. The full quality appraisal checklists are available in Additional File 4.

### Mapping recent implementation literature to the quality implementation Framework

An overview of the QIF [15] phases and steps present in the included articles, including the percentage of articles addressing each step, is available in Table 2. Table 2 presents the deductive mapping of each included article against the 14 QIF steps. A binary coding approach present (+)/not present (-) was applied to indicate whether each step was explicitly or substantively addressed in the review.

**Table 2:** The quality implementation Framework [15] phases and steps present* in the included articles

QIF phases and steps	**Albers** [32]	**Chaudoir** [33]	**Escoffery** [34]	**Huybrechts** [35]	**Li** [36]	**Mahmoud** [37]	**McHugh** [38]	**Nair** [39]
*Phase One: Initial considerations* 1. Needs and resources assessment 2. Fit assessment 3. Capacity/readiness assessment 4. Possibility of adaptation 5. Buy-in; supportive climate 6. General org. capacity building 7. Staff recruitment/maintenance 8. Pre-innovation training *Phase Two: Structure for implementation* 9. Implementation teams 10. Implementation plan *Phase Three: Ongoing support strategies* 11. TA/coaching/supervision 12. Process evaluation 13. Feedback mechanism *Phase Four: Improving future applications* 14. Learning from experience	+ + + - + + + + + + + - - -	+ + + + + + + + + - + + + +	+ + + + + + + + + - + - + -	+ + + + - + - - + + + + + -	+ - + - + + + + + + + + + +	+ + + + + + + + - + + + - +	+ + - + + - + + - - - - - +	+ + + + + + + + + + + + + +
**QIF phases and steps**	**Nilsen** [40]	**Ostermeier** [41]	**Piat** [42]	**Richter** [43]	**Shierk** [44]	**Toms** [45]	**Varsi** [46]	**Articles reporting, %**
*Phase One: Initial considerations* 1. Needs and resources assessment 2. Fit assessment 3. Capacity/readiness assessment 4. Possibility of adaptation 5. Buy-in; supportive climate 6. General org. capacity building 7. Staff recruitment/maintenance 8. Pre-innovation training *Phase Two: Structure for implementation* 9. Implementation teams 10. Implementation plan *Phase Three: Ongoing support strategies* 11. TA/coaching/supervision 12. Process evaluation 13. Feedback mechanism *Phase Four: Improving future applications* 14. Learning from experience	+ - + + + + + + + + + + + +	+ + + + + - + + - - + + - +	+ + + + + + + + + + + - - -	+ + - + + + + + + + + + + -	+ + + + + - - - - + - - - +	+ + + + + + + + + + + - - -	+ + + + + + + + + + - + + +	100 87 87 87 93 80 87 87 73 73 80 60 53 60

*+ = present (step explicitly or substantively addressed). - = not present

## Phase one: initial considerations regarding the host setting

### Assessment strategies

#### Step 1. Conducting a needs and resources assessment

Pre-implementation activities such as exploration and situational analysis are foundational, aiming to uncover local needs, contextual challenges, and readiness conditions [32, 35, 41]. Key considerations include resource availability - financial, technological, infrastructural - and evaluation of staff capacity and logistical factors like facilities and equipment [33, 36, 37, 40, 42, 43]. Legal, ethical, and regulatory contexts are also critical, especially regarding information and communication technology (ICT) requirements, privacy standards, and reimbursement policies [33, 34, 37]. Systematic appraisal of existing evidence and data-driven analysis of the magnitude and nature of the problem are also important elements [39]. Broader economic conditions and external policy influences further shape implementation feasibility [−34, 38, 46]. Incorporating local knowledge and end-user preferences ensures cultural appropriateness and alignment of innovations with identified needs [43–45].

#### Step 2. Conducting a fit assessment

Assessing the compatibility of innovations with organizational workflows, culture, and staff attitudes is emphasized [32, 33, 35, 37, 41, 42]. Compatibility extends to alignment with organizational mission, team dynamics, and communication practices [33]. Innovation design quality, packaging, and goal alignment related to service users’ needs are also crucial [43–45]. External policy and regulatory environments, including ethical compliance and reimbursement structures, influence fit [34–37]. Assessing fit further includes evaluation of data relevance, algorithmic bias, regulatory certification, and compatibility with existing digital infrastructures and vendor agreements [39]. Patient needs and preferences underline the importance of tailoring innovations to local populations and cultures [38, 45]. Iterative evaluative strategies facilitate ongoing refinement of this fit during early implementation phases [46].

#### Step 3. Conducting a capacity/readiness assessment

Readiness assessments address both general and innovation-specific capacities, including leadership, climate, staffing, skills, and prior experience [32, 33, 36, 37, 40, 45]. Psychological readiness encompasses motivation, enthusiasm, and self-efficacy concerning evidence-based practices [33, 40]. Workforce factors such as workload and licensure, alongside organizational culture and leadership styles, influence readiness [32, 33, 36, 37, 40, 45]. Broader cultural and policy contexts, including mandates and community norms, also affect systemic preparedness [34, 42]. ICT infrastructure and reimbursement mechanisms are crucial for sustaining adoption [37, 46]. This includes assessing whether the existing technical environment and computing capacity are sufficient to support the innovation, as well as considering potential changes in staff roles and competencies [39]. General exploration and situational analyses complement readiness by appraising internal capabilities and change impetus [35, 41, 44].

### Decisions about adaptation

Step 4. Possibility for adaptation

The capacity to adapt innovations without compromising core elements is critical, involving attributes such as adaptability, complexity, trialability, and observability [33]. Examples include language adjustments, aligning training content, and modifying service delivery to local needs [37, 38, 43–45]. Cultural competence and patient-centered goal fit should guide appropriate adaptations [37, 42, 45]. Legal and policy frameworks may constrain or mandate modifications [34, 35, 40]. Adaptation may also involve local pilot testing and modification of workflows, digital infrastructures, and contractual arrangements to ensure feasibility within specific organizational settings [39]. Iterative evaluative strategies support adaptation while preserving fidelity [41, 46]. Adaptation is thus a strategic asset enhancing contextual fit.

### Capacity-building strategies

#### Step 5. Obtaining explicit buy-in from critical stakeholders and fostering a supportive community/organizational climate

Commitment from stakeholders across levels - from frontline providers to policymakers - is vital for alignment and ownership [32, 42, 43]. Early and ongoing engagement fosters trust, relevance, and momentum [42, 46]. Collaboration, teamwork, communication, and local champions enhance buy-in [36, 40, 44, 45]. Leadership and organizational culture facilitate receptivity and psychological safety [37, 40, 45]. Addressing concerns related to legal responsibility, transparency, safety, and explainability is also important for securing stakeholder commitment and organizational support [39]. Partnerships beyond the organization expand legitimacy, resources, and mutual learning [34, 38, 41, 43]. Interprofessional networks and equity-focused strategies promote inclusion and shared goals [34, 41, 46]. Policymaker endorsement signals legitimacy and enables financial and regulatory support [33, 34].

#### Step 6. Building general/organizational capacity

Strengthening organizational capacity involves leadership, communication, supportive team structures and alignment of workflows and internal relationships [32, 36, 39, 40, 45]. Stable infrastructure is essential for sustained implementation [33, 35, 37]. Cultivating a culture of trust, openness, and collaboration shapes the implementation environment [34, 43, 45]. Leadership aligns expectations and strategic planning [36, 40, 45]. External relationships and professional networks facilitate resource access and legitimacy [32, 34]. Staffing adequacy, time availability, licensure, and competencies denote readiness [36, 37, 42]. Iterative learning strategies involving feedback and reflection support capacity building [46].

#### Step 7. Staff recruitment/maintenance

Recruitment of qualified personnel and identification of change agents are critical [32, 41, 46]. Utilizing existing networks aids in securing committed staff [43]. Staff stability and engagement depend on supportive leadership, role clarity, and positive work environments [33, 36, 39, 45]. Team spirit, collaboration, and communication underpin capacity and reduce turnover [36, 40, 45]. Workload, job satisfaction, and emotional exhaustion affect staff retention and performance [33, 42]. Personnel qualifications, including licensure and cultural competence, ensure fidelity [37]. Peer norms and professional identity further influence recruitment and engagement [34, 38].

#### Step 8. Effective pre-innovation staff training

Structured training ensures knowledge, skills, and attitudes for innovation delivery [32, 36, 39, 41, 45]. Tailoring training content to local contexts enhances relevance [42, 43]. Organizational learning strategies include continuous reflection and knowledge sharing [33, 46]. Change agents facilitate bridging innovation and practice [32, 41]. Interprofessional learning and alignment with shared visions promote coherence [34, 38]. Licensure, prior experience, and cultural competence remain important for specialized or sensitive settings [37]. Competent and confident staff enable smooth implementation transitions [40, 45].

## Phase two: creating a structure for implementation

### Structural features for implementation

#### Step 9. Creating implementation teams

Implementation teams coordinate efforts, span organizational boundaries, and align activities with goals [32, 35, 36]. Multiple teams may be required based on complexity [32]. Effective teams demonstrate collaboration, shared leadership, clear roles and responsibilities, and champion individuals [36, 39, 45]. Team capacity and team spirit, commitment and communication determine success [33, 45]. Organizational culture, leadership, and social relationships support team functioning [40, 42, 43]. Engaging staff from key organizational sectors anchors implementation in daily practice [43]. Peer norms and infrastructure shape team responsibilities and function [33, 34, 46].

#### Step 10. Developing an implementation plan

Clear, structured implementation plans guide innovation operationalization, including team coordination and resource alignment [32, 35]. Tailoring plans to context and population involves adapting content, training, and monitoring tools [37, 43–45]. Planning activities include standard operating procedures, training needs identification, outcome measures, monitoring systems, communication strategies, and assignment of organizational ownership [37, 39]. Administrative infrastructures support logistics and engagement [36, 45]. Planning is iterative, allowing responsiveness to challenges as they arise [46]. Reconciling differing paradigms, such as biomedical versus recovery-oriented approaches, aids coherent implementation [42]. Leadership and support enable implementation planning [36, 40].

## Phase three: ongoing structure once implementation begins

### Ongoing implementation support strategies

#### Step 11. Technical assistance/coaching/supervision

Continuous technical assistance and supervisory support enable successful implementation [32, 37, 45]. Support includes troubleshooting, guidance, and access to expertise, IT support, and availability of relevant specialists and resources [35, 36, 39, 43]. Pre-testing technologies, and role clarity facilitate complex implementations [37, 39]. Organizational encouragement, team problem-solving, and interpersonal support foster adaptability [33, 40, 42]. Communication infrastructure and external networks enhance expertise sharing [34, 41]. Support must be flexible, continuous, and responsive to challenges.

#### Step 12. Process evaluation

Evaluating implementation processes is essential [33, 35, 36, 46]. Continuous monitoring, feedback loops, and iterative adjustments improve fidelity and responsiveness including assessment of system use, workflow impact, and unintended consequences [36, 39, 40, 46]. Process evaluation ensures compliance with contextual norms and guides adaptations [37, 43]. Leadership support and shared decision-making foster learning environments [33]. Though some contributions focus on phases or program management [32, 41], these support dynamic, feedback-informed strategies. Systematic process evaluation identifies effective practices under varying conditions.

#### Step 13. Supportive feedback mechanism

Supportive feedback loops enable continuous learning and practice refinement [36, 40, 43, 46]. Feedback is embedded within organizational cultures of reflection and adaptability [33, 40]. Leadership encouragement and team learning maintain motivation and direction [4243]. Communication infrastructures and professional networks facilitate feedback exchange, supporting identification of risks, needs for adaptation, and additional training, especially in complex settings [34, 35, 39]. Though sometimes informal, these mechanisms foster knowledge sharing and participant responsiveness, ensuring implementation remains contextually grounded.

### Phase four: improving future applications

#### Step 14. Learning from experience

Capturing and applying lessons learned is vital for future improvements [33, 37, 46]. Mechanisms include audits, peer reviews, and monitoring cost-efficiency, staff satisfaction, and technical performance [37]. Embedded learning cycles use experiences to inform subsequent implementation stages [44, 46]. Tracking fidelity, effectiveness, sustainability, and ongoing system performance supports retrospective and continuous organizational learning [39, 41]. Feedback and monitoring contribute when linked to longer-term reflection and adaptation [36, 40]. Deliberate learning strengthens local practices and advances IS knowledge.

## Additional elements beyond the quality implementation Framework in recent literature

Literature published since the development of QIF has introduced additional themes that complement and expand on the original structure. The present synthesis identified three overarching themes that extend beyond the scope of the original four phases and 14 steps:

1. The importance of foundational evidence and preparatory knowledge.
2. The need to explicitly include sustainability as a separate phase.
3. The relevance of integrating key cross-cutting elements throughout the implementation process, including the roles of service users, intervention deliverer, context and technology.

Several of the included articles emphasized that implementation efforts should be grounded in a structured understanding of existing evidence prior to engaging in active implementation planning. This includes evidence generation, validation, and appraisal, as well as translation of findings into contextually relevant practice guidelines. This was further supported by Nair et al., who emphasised the role of systematic evidence appraisal and data-driven problem identification as part of early implementation considerations [39]. Huybrechts et al. outlined a sequence of pre-implementation activities that included evidence summarisation, feasibility testing, grading of evidence, and translation into actionable guidelines. All activities that precede and inform capacity assessments or planning stages [35]. Similarly, Chaudoir et al. highlighted the organizational value placed on research involvement, knowledge acquisition, and internal research competence as foundational to successful implementation [33]. These preparatory elements are not explicitly represented in the original QIF but appear central in shaping the readiness and appropriateness of subsequent implementation efforts.

Toms et al. also pointed to the significance of clearly understanding the evidence base and the characteristics of the intervention before initiating implementation planning [45]. These findings underscore the importance of an initial step focused on evidence and innovation appraisal, including the degree of clarity, feasibility, usability, and adaptability of the innovation in question [33, 37, 43].

While step 14 of QIF addresses learning from experience, several articles suggest that sustainability involves a more active and ongoing set of practices that warrants recognition as a separate phase. The concept of sustainability was repeatedly emphasised as both an endpoint and a continuous process requiring specific strategies, such as long-term resource planning, adaptation maintenance, repeated training, infrastructure support, regulatory alignment, ongoing monitoring of system performance and governance mechanisms [32, 37, 39, 46].

Huybrechts et al. described a post-implementation sustainability phase involving institutionalisation, diffusion, and integration into routine practice [35]. Mahmoud et al. provided further granularity, identifying activities such as maintaining licensure and certification, refining service delivery, and ensuring adherence to evolving policy frameworks [37]. Varsi et al. introduced sustainability as one of several key implementation outcomes, alongside feasibility, acceptability, adoption, and fidelity [46]. Collectively, these findings support the need for a distinct sustainability-oriented phase that captures the dynamic, long-term work of embedding innovations into routine care.

A prominent theme across several included articles was the importance of explicitly considering and integrating multiple intersecting perspectives throughout the implementation process. These included characteristics and needs of the patient or service user [37, 38, 42], contextual influences at both organizational and system levels, including governance structures, workflow integration, and organizational processes [33, 34, 39, 40], technological requirements and readiness, including data infrastructure, system integration, and ongoing performance monitoring [37, 39] and provider characteristics and roles [36, 45].

In particular, the role of the patient and the broader community was frequently cited as central to implementation success. This encompassed needs, preferences, satisfaction, engagement, and outcomes as well as the need to tailor interventions to individual motivation, engagement, and everyday contexts [34, 37, 42, 44], which were often not fully addressed within the existing QIF steps. Likewise, the role of the provider, including attitudes, competence, active engagement, and the ability to tailor and deliver interventions in practice, as well as the presence of key individuals such as change agents or champions, was highlighted as a recurrent determinant of successful implementation [33, 36, 44].

Mahmoud et al. proposed that implementation should be tailored and repeated across multiple dimensions, including the patient, provider, technological infrastructure, and contextual setting [37]. This was echoed by Albers et al., who suggested dividing implementation into multiple socio-ecological levels, each of which may require differentiated strategies [32]. Chaudoir et al. and Huybrechts et al. further elaborated that these elements function not as discrete inputs at specific timepoints, but as continuously interacting influences throughout implementation [33, 35]. Nair et al. also emphasize the importance of integrating technological infrastructure, workflow adaptation, and continuous system monitoring as ongoing and interacting components throughout the implementation process [39].

## From literature to practice: towards an updated QIF model for future implementation

**Fig. 3:**
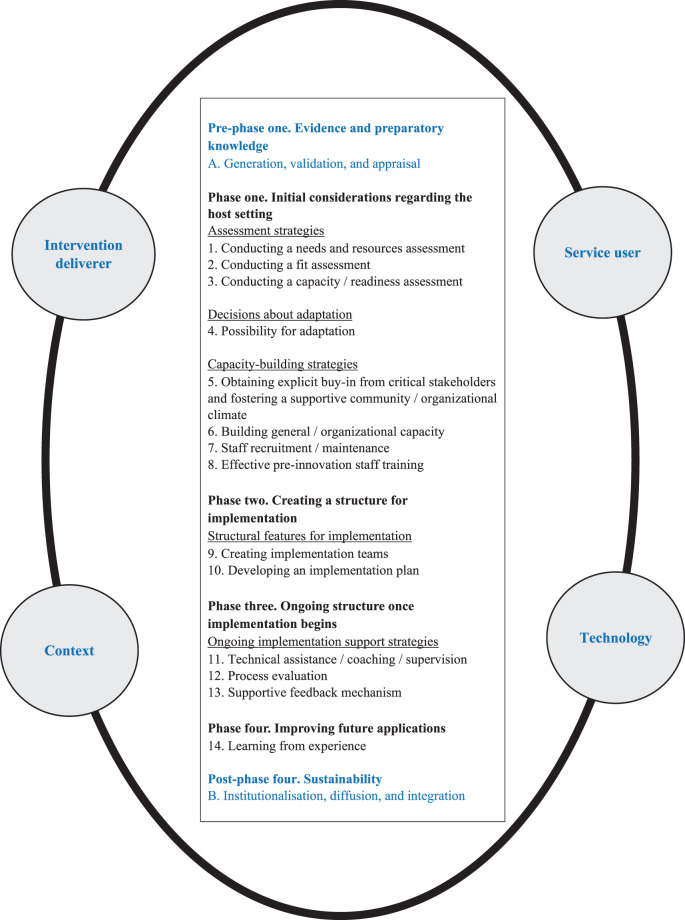
The updated quality implementation framework

Based on our synthesis, we propose an updated version of QIF that better aligns with current IS evidence and practice (Fig. 3). The model introduces a new pre-phase one focused on evidence and preparatory knowledge and adds a new post-phase four focused on sustainability, thereby extending the scope beyond the original four phases and 14 steps. Pre-phase one addresses the need for structured appraisal, validation, and contextualisation of evidence before implementation begins. This phase lays the foundation for ensuring that innovations are feasible, appropriate, and aligned with the setting. Post-phase four recognises sustainability as an active, ongoing process requiring strategies for institutionalisation, integration, and long-term adaptability. In addition, the updated QIF model integrates four core domains, service user, intervention deliverer, context, and technology – which we propose to apply as systematic lenses across all phases and steps of implementation. Rather than being treated as background conditions, each domain should be explicitly examined throughout the framework. For instance, implementers can trace how service users perspectives are integrated during initial assessments, adaptation processes, training design, and learning mechanisms. Similarly, the intervention deliverer domain can be followed through assessments of readiness, role clarity, and ongoing support, the context domain through organizational culture, leadership, and external systems, and the technology domain through evaluation of usability, integration, and sustainability strategies. By applying each domain consistently across the full implementation process, the updated QIF provides practical guidance on *how to* implement in complex real-world settings and offers a structured yet flexible model that supports more tailored, operationally relevant, and sustainable implementation efforts. In Meyers et al. [15], the critical phases and steps were accompanied by key questions intended to support the implementation process. These questions, along with additional questions developed for the two additional phases introduced in this study, are available in Table 3.

**Table 3:** Critical steps in implementation and important questions to answer at each step in the quality implementation Framework as developed and published by Meyers et al. In 2012 [15] and additional phases and steps included in Fig. 3 in the current study

**Pre-phase one. Evidence and preparatory knowledge** A. Generation, validation, and appraisal What is the source of the evidence supporting the innovation? Has the evidence been critically appraised and validated? Are there gaps in the current evidence base that require further consideration?
**Phase one: Initial considerations regarding the host setting** Assessment strategies *1. Conducting a needs and resources assessment:* Why are we doing this? What problems or conditions will the innovation address (i.e., the need for the innovation)? What part(s) of the organization and who in the organization will benefit from improvement efforts?
*2. Conducting a fit assessment:* Does the innovation fit the setting? How well does the innovation match the: Identified needs of the organization/community? Organization’s mission, priorities, values, and strategy for growth? Cultural preferences of groups/consumers who participate in activities/services provided by the organization/community?
*3. Conducting a capacity/readiness assessment:* Are we ready for this? To what degree does the organization/community have the will and the means (i.e., adequate resources, skills and motivation) to implement the innovation? Is the organization/community ready for change?
Decisions about adaptation *4. Possibility for adaptation:* Should the planned innovation be modified in any way to fit the host setting and target group? What feedback can the host staff offer regarding how the proposed innovation needs to be changed to make it successful in a new setting and for its intended audience? How will changes to the innovation be documented and monitored during implementation?
Capacity Building Strategies (may be optional depending on the results of previous elements) *5. Obtaining explicit buy-in from critical stakeholders and fostering a supportive community/organizational climate:* Do we have genuine and explicit buy-in for this innovation from: Leadership with decision-making power in the organization/community? From front-line staff who will deliver the innovation? The local community (if applicable)? Have we effectively dealt with important concerns, questions, or resistance to this innovation? What possible barriers to implementation need to be lessened or removed? Can we identify and recruit an innovation champion(s)? Are there one or more individuals who can inspire and lead others to implement the innovation and its associated practices? How can the organization/community assist the champion in the effort to foster and maintain buy-in for change? *Note.* Fostering a supportive climate is also important after implementation begins and can be maintained or enhanced through such strategies as organizational policies favoring the innovation and providing incentives for use and disincentives for non-use of the innovation
*6. Building general/organizational capacity:* What infrastructure, skills, and motivation of the organization/community need enhancement in order to ensure the innovation will be implemented with quality? Of note is that this type of capacity does not directly assist with the implementation of the innovation, but instead enables the organization to function better in a number of its activities (e.g., improved communication within the organization and/or with other agencies; enhanced partnerships and linkages with other agencies and/or community stakeholders).
*7. Staff recruitment/maintenance:* Who will implement the innovation? Initially, those recruited do not necessarily need to have knowledge or expertise related to use of the innovation; however, they will ultimately need to build their capacity to use the innovation through training and on-going support Who will support the practitioners who implement the innovation? These individuals need expertise related to (a) the innovation, (b) its use, (c) implementation science, and (d) process evaluation so they can support the implementation effort effectively Might roles of some existing staff need realignment to ensure that adequate person-power is put towards implementation?
*8. Effective pre-innovation staff training:* Can we provide sufficient training to teach the why, what, when, where, and how regarding the intended innovation? How can we ensure that the training covers the theory, philosophy, values of the innovation, and the skill-based competencies needed for practitioners to achieve self-efficacy, proficiency, and correct application of the innovation?
**Phase two: Creating a structure for implementation** Structural features for implementation *9. Creating implementation teams:* Who will have organizational responsibility for implementation? Can we develop a support team of qualified staff to work with front-line workers who are delivering the innovation? Can we specify the roles, processes, and responsibilities of these team members?
*10. Developing an implementation plan:* Can we create a clear plan that includes specific tasks and timelines to enhance accountability during implementation? What challenges to effective implementation can we foresee that we can address proactively?
**Phase three: Ongoing structure once implementation begins** Ongoing implementation support strategies *11. Technical assistance/coaching/supervision:* Can we provide the necessary technical assistance to help the organization/community and practitioners deal with the inevitable practical problems that will develop once the innovation begins? These problems might involve a need for further training and practice in administering more challenging parts of the innovation, resolving administrative or scheduling conflicts that arise, acquiring more support or resources, or making some required changes in the application of the innovation
*12. Process evaluation:* Do we have a plan to evaluate the relative strengths and limitations in the innovation’s implementation as it unfolds over time? Data are needed on how well different aspects of the innovation are being conducted as well as the performance of different individuals implementing the innovation
*13. Supportive feedback mechanism:* Is there an effective process through which key findings from process data related to implementation are communicated, discussed, and acted upon? How will process data on implementation be shared with all those involved in the innovation (e.g., stakeholders, administrators, implementation support staff, and front-line practitioners)? This feedback should be offered in the spirit of providing opportunities for further personal learning and skill development and organizational growth that leads to quality improvement in implementation
**Phase four: Improving future applications** *14. Learning from experience:* What lessons have been learned about implementing this innovation that we can share with others who have an interest in its use? Researchers and innovation developers can learn how to improve future implementation efforts if they critically reflect on their experiences and create genuine collaborative relationships with those in the host setting Collaborative relationships appreciate the perspectives and insights of those in the host setting and create open avenues for constructive feedback from practitioners on such potentially important matters as: (a) the use, modification, or application of the innovation; and (b) factors that may have affected the quality of its implementation
**Post-phase four. Sustainability** *B. Institutionalisation, diffusion, and integration* Has the innovation been adopted into everyday practice? What ongoing organisational and cultural structures have been established to maintain the innovation over time? Has the innovation diffused beyond the initial context?

## Discussion

This study aimed to update the QIF model by synthesizing findings from published reviews on implementation TMFs since 2012. The resulting update introduces a pre-phase focused on evidence appraisal and a post-phase focused on sustainability, alongside cross-cutting domains that account for service user, intervention deliverer, context, and technology. Our findings propose that QIF continues to be a relevant and practically useful process model, with recent literature supporting the importance of its core phases and elements. However, evolving implementation complexities call for an expanded conceptualization of the implementation process. Our proposal for an updated QIF adds value to the existing literature in several ways. It systematically appraises whether the foundational phases and steps of QIF continue to reflect the realities of implementation in healthcare and social science. It translates these findings into an updated model. And it offers a solution for bridging the persistent gap between theoretical knowledge and actionable guidance.

A major strength of QIF lies in its ability to guide implementation in real-world settings [15]. Process models such as QIF are especially valued by practitioners for their structured, phase-based design and their direct applicability to daily practice [1–3, 15, 39]. This may help explain the growing popularity of QIF across Scandinavian countries, where structured implementation approaches are frequently embedded in public sector initiatives concerning healthcare and social science [20–22]. However, despite its practical relevance, QIF and other process models remain relatively underutilized in implementation research compared to determinant frameworks or theoretical models. This lack of alignment raises important questions about the relationship between IS and implementation practice. Process models are often seen as operational tools rather than contributions to theory. This perception may underestimate TMFs that support the *how to* of implementation despite their high uptake in practice. This calls for a stronger focus on legitimizing process models as a serious and essential component in the IS toolbox in the future.

Some limitations of this study should be noted. Although the search strategy was comprehensive and included four major databases, it was limited to English-language articles. This may have excluded relevant literature, particularly from non-English-speaking regions such as Latin America, Asia, and parts of Europe, where implementation research may be published in local languages and reflect diverse contextual and cultural perspectives. While the decision to restrict the study to English-language articles was a pragmatic, methodological decision, it likely introduced a bias towards studies conducted in high-income countries and may have led to an underrepresentation of regionally important contributions.

While a broad range of review types were included, the heterogeneity of review aims, methods, and the types of TMFs used introduced challenges in synthesis and comparability. One possible alternative approach could have been to restrict inclusion to articles that explicitly applied process models. While this might have increased comparability, it would also have significantly narrowed the evidence base and excluded potentially relevant insights on implementation processes drawn from other types of TMFs. A way to expand the evidence base of process models in future studies could be to include primary empirical studies, which may offer more insights into how implementation processes are applied in practice. In the present study, however, we chose to focus on reviews to provide a structured overview of how TMFs have been applied across existing implementation literature. The chosen inclusion criteria allowed for a broader view of how implementation is described, conceptualized and applied in the literature, but it also required careful interpretation in mapping findings to QIF. To strengthen the mapping, two reviewers independently mapped extracted data to the QIF framework and subsequently resolved discrepancies through discussion and consensus. While this procedure supports consistency, it is important to acknowledge that the process inherently involved interpretive judgement. Given that the data were qualitative and narrative in nature rather than numeric or standardized, some subjectivity in interpretation was unavoidable.

Future research should prioritize testing and refining the updated QIF in empirical research and daily practice. Process models should also be more deliberately integrated and actively used when designing and planning implementation. This study contributes to the field by offering evidence-based updates to a widely used framework, responding to emerging needs in implementation science and practice. By expanding the original scope of QIF and incorporating cross-cutting domains, the updated model offers more explicit guidance for practitioners and a more comprehensive structure for researchers. A more intentional use of process models may support efforts to narrow the gap between knowledge and action.

## Conclusions

This study synthesized findings from published reviews to update the QIF. The updated framework introduces two new phases concerning evidence and preparatory knowledge, and sustainability and integrates four cross-cutting domains (service user, intervention deliverer, context, technology). The additions reflect the emerging complexity in implementation and give practical guidance on the *how to* in real-world settings. By updating QIF according to new evidence, this study strengthens the framework as a tool of value both in IS and implementation practice, supporting a more comprehensive, up-to-date approach to future implementation in healthcare and social science.

## Electronic supplementary material

Below is the link to the electronic supplementary material.

Supplementary Material 1

Supplementary Material 2

Supplementary Material 3

Supplementary Material 4

## Data Availability

All data generated or analyzed during this study are included in this published article and its supplementary information files.
