# Supplementary material for: Updating the critical steps of the quality implementation framework: an umbrella review of reviews
Source: Implement Sci Commun. 2026 May 27;7:112. doi: 10.1186/s43058-026-00954-7 (PMC13242111; doi:10.1186/s43058-026-00954-7)
Supplement: Supplementary file 2 — Supplementary Material 2 [file 43058_2026_954_MOESM2_ESM.docx]

Search history

Search Update

Updating the critical steps of the Quality Implementation Framework: an umbrella review of reviews

Databases and results

| Database | Platform | Result | Date |
| --- | --- | --- | --- |
| PubMed | PubMed.gov | 1264 | 30.01.2026 |
| PsycINFO | APA | 126 | 30.01.2026 |
| Scopus | Scopus.com | 1067 | 30.01.2026 |
| Web of Science | Wiley | 567 | 30.01.2026 |

PubMed

| Search | Query | Results |
| --- | --- | --- |
| #4 | Search: (((("implementation"[Title] OR "determinants of implementation"[Title/Abstract] OR "implementation science"[Title/Abstract] OR "implementation-effectiveness"[Title/Abstract] OR "implementation evaluation"[Title/Abstract] OR "implementation determinant*"[Title/Abstract] OR "implementation outcome*"[Title/Abstract] OR "implementation science"[MeSH Terms]) AND ("framework*"[Title/Abstract] OR "model*"[Title/Abstract] OR "tool*"[Title/Abstract] OR "implementation framework*"[Title/Abstract] OR "consolidated framework for implementation research"[Title/Abstract] OR "framework synthesis"[Title/Abstract]) AND ("review*"[Title/Abstract] OR "systematic review*"[Title/Abstract] OR "review literature as topic"[MeSH Terms] OR "systematic reviews as topic"[MeSH Terms] OR "meta analysis as topic"[MeSH Terms]))) AND (English[Language])) AND (("2025/01/01"[Date - Publication] : "2026"[Date - Publication])) Sort by: Publication Date | [1,264](https://pubmed.ncbi.nlm.nih.gov/?term=%28%28%28%28%22implementation%22%5BTitle%5D+OR+%22determinants+of+implementation%22%5BTitle%2FAbstract%5D+OR+%22implementation+science%22%5BTitle%2FAbstract%5D+OR+%22implementation-effectiveness%22%5BTitle%2FAbstract%5D+OR+%22implementation+evaluation%22%5BTitle%2FAbstract%5D+OR+%22implementation+determinant%2A%22%5BTitle%2FAbstract%5D+OR+%22implementation+outcome%2A%22%5BTitle%2FAbstract%5D+OR+%22implementation+science%22%5BMeSH+Terms%5D%29+AND+%28%22framework%2A%22%5BTitle%2FAbstract%5D+OR+%22model%2A%22%5BTitle%2FAbstract%5D+OR+%22tool%2A%22%5BTitle%2FAbstract%5D+OR+%22implementation+framework%2A%22%5BTitle%2FAbstract%5D+OR+%22consolidated+framework+for+implementation+research%22%5BTitle%2FAbstract%5D+OR+%22framework+synthesis%22%5BTitle%2FAbstract%5D%29+AND+%28%22review%2A%22%5BTitle%2FAbstract%5D+OR+%22systematic+review%2A%22%5BTitle%2FAbstract%5D+OR+%22review+literature+as+topic%22%5BMeSH+Terms%5D+OR+%22systematic+reviews+as+topic%22%5BMeSH+Terms%5D+OR+%22meta+analysis+as+topic%22%5BMeSH+Terms%5D%29%29%29+AND+%28English%5BLanguage%5D%29%29+AND+%28%28%222025%2F01%2F01%22%5BDate+-+Publication%5D+%3A+%222026%22%5BDate+-+Publication%5D%29%29&sort=pubdate&size=200&ac=no) |

PsycINFO

| Search | Query | Results |
| --- | --- | --- |
| #1 | (((Any Field: AnyFieldFilt: (Any Field: "Literature Review")) OR (title: ("review*") OR title: ("systematic review*")) OR (abstract: ("review*") OR abstract: ("systematic review*")))) AND (((title: ("framework*") OR title: ("model*") OR title: ("tool*") OR title: ("implementation framework*") OR title: ("consolidated framework for implementation research") OR title: ("framework synthesis")) OR (abstract: ("framework*") OR abstract: ("model*") OR abstract: ("tool*") OR abstract: ("implementation framework*") OR abstract: ("consolidated framework for implementation research") OR abstract: ("framework synthesis")))) AND (((title: ("implementation") OR title: ("determinants of implementation") OR title: ("implementation science") OR title: ("implementation-effectiveness") OR title: ("implementation evaluation") OR title: ("implementation determinant*") OR title: ("implementation outcome*")) OR (abstract: ("determinants of implementation") OR abstract: ("implementation science") OR abstract: ("implementation-effectiveness") OR abstract: ("implementation evaluation") OR abstract: ("implementation determinant*") OR abstract: ("implementation outcome*")))) AND Year: 2025 To 2026  Search Databases: APA PsycInfo , APA PsycArticles , APA PsycTests , APA PsycTherapy | 126 |

Scopus

| # | Queries | Results |
| --- | --- | --- |
| #1 | (TITLE-ABS-KEY("quality implementation framework" OR ("implementation" W/2 "model*") OR ("implementation" W/2 "framework*") OR ("implementation*" W/2 "tool*") OR "implementation evaluation" OR "determinants of implementation" OR "implementation science" OR "implementation effectiveness" OR "implementation evaluation" OR "implementation determinant*" OR "implementation outcome*" OR ("implementation science" W/2 "model*") OR ("implementation science" W/2 "framework*") OR ("implementation* science" W/2 "tool*")) AND TITLE-ABS-KEY("review")) AND PUBYEAR > 2024 AND PUBYEAR < 2027 AND ( LIMIT-TO ( DOCTYPE,"re" ) ) AND ( LIMIT-TO ( LANGUAGE,"English" ) ) | 1067 |

Web of Science

| # | Query | Results |
| --- | --- | --- |
| #1 | "implementation" OR "determinants of implementation" OR "implementation science" OR "implementation-effectiveness" OR "implementation evaluation" OR "implementation determinant*" OR "implementation outcome*" (Title) or "determinants of implementation" OR "implementation science" OR "implementation-effectiveness" OR "implementation evaluation" OR "implementation determinant*" OR "implementation outcome*" (Abstract)  AND  "framework*" OR "model*" OR "tool*" OR "implementation framework*" OR "consolidated framework for implementation research" OR "framework synthesis" (Title) or "framework*" OR "model*" OR "tool*" OR "implementation framework*" OR "consolidated framework for implementation research" OR "framework synthesis" (Abstract)  AND  "review*" OR "systematic review*" (Title) and "review*" OR "systematic review*" (Abstract)  AND  English (Languages) AND Timespan: 2025-01-01 to 2026-02-01 (Index Date) | 567 |

# Matrix

| Implementation | Framework | Review |
| --- | --- | --- |
| implementation (Title) OR  determinants of implementation (Title/abstract) OR  implementation science (Title/abstract) OR  implementation-effectiveness (Title/abstract) OR  implementation evaluation (Title/abstract) OR  implementation determinant* (Title/abstract) OR  Implementation outcome* (Title/abstract) OR  implementation science (MeSH) | framework* (Title/abstract) OR  model* (Title/abstract) OR  tool* (Title/abstract) OR  implementation framework* (Title/abstract) OR  consolidated framework for implementation research (Title/abstract) OR  framework synthesis (Title/abstract) | review* (Title/abstract) OR  systematic review* (Title/abstract) OR  Review literature as topic (MeSH) OR  Systematic reviews as topic (MeSH) OR  Meta-analysis as topic (MeSH) |

# MEDLINE PubMed

17^th^ January 2025

**Search: (("implementation"[Title] OR "determinants of implementation"[Title/Abstract] OR "implementation science"[Title/Abstract] OR "implementation-effectiveness"[Title/Abstract] OR "implementation evaluation"[Title/Abstract] OR "implementation determinant*"[Title/Abstract] OR "implementation outcome*"[Title/Abstract] OR "implementation science"[MeSH Terms]) AND ("framework*"[Title/Abstract] OR "model*"[Title/Abstract] OR "tool*"[Title/Abstract] OR "implementation framework*"[Title/Abstract] OR "consolidated framework for implementation research"[Title/Abstract] OR "framework synthesis"[Title/Abstract]) AND ("review*"[Title/Abstract] OR "systematic review*"[Title/Abstract] OR "review literature as topic"[MeSH Terms] OR "systematic reviews as topic"[MeSH Terms] OR "meta analysis as topic"[MeSH Terms])) AND ((2023/10/01:2024/12/31[pdat]) AND (english[Filter])) Sort by: Most Recent**

("implementation"[Title] OR "determinants of implementation"[Title/Abstract] OR "implementation science"[Title/Abstract] OR "implementation-effectiveness"[Title/Abstract] OR "implementation evaluation"[Title/Abstract] OR "implementation determinant*"[Title/Abstract] OR "implementation outcome*"[Title/Abstract] OR "implementation science"[MeSH Terms]) AND ("framework*"[Title/Abstract] OR "model*"[Title/Abstract] OR "tool*"[Title/Abstract] OR "implementation framework*"[Title/Abstract] OR "consolidated framework for implementation research"[Title/Abstract] OR "framework synthesis"[Title/Abstract]) AND ("review*"[Title/Abstract] OR "systematic review*"[Title/Abstract] OR "review literature as topic"[MeSH Terms] OR "systematic reviews as topic"[MeSH Terms] OR "meta analysis as topic"[MeSH Terms]) AND (2023/10/01:2024/12/31[Date - Publication] AND "english"[Language])

**Translations**

**english[Filter]: english [LA]**

-------------------------------------

Search 25^th^ of October 2023

## History and Search Details

Øverst på formularen

| **Search** | **Actions** | **Details** | **Query** | **Results** | **Time** |
| --- | --- | --- | --- | --- | --- |
| #6 |  |  | Search: **(("implementation"[Title] OR "determinants of implementation"[Title/Abstract] OR "implementation science"[Title/Abstract] OR "implementation-effectiveness"[Title/Abstract] OR "implementation evaluation"[Title/Abstract] OR "implementation determinant*"[Title/Abstract] OR "implementation outcome*"[Title/Abstract] OR "implementation science"[MeSH Terms]) AND ((((((framework*[Title/Abstract]) OR (model*[Title/Abstract])) OR (tool*[Title/Abstract])) OR (implementation framework*[Title/Abstract])) OR ("consolidated framework for implementation research"[Title/Abstract])) OR ("framework synthesis"[Title/Abstract]))) AND (((((review*[Title/Abstract]) OR (systematic review*[Title/Abstract])) OR (Review literature as topic[MeSH])) OR (Systematic reviews as topic[MeSH])) OR (Meta-analysis as topic[MeSH]))** Filters: **English, from 2012 - 3000/12/12** Sort by: **Most Recent**  (("implementation"[Title] OR "determinants of implementation"[Title/Abstract] OR "implementation science"[Title/Abstract] OR "implementation-effectiveness"[Title/Abstract] OR "implementation evaluation"[Title/Abstract] OR "implementation determinant*"[Title/Abstract] OR "implementation outcome*"[Title/Abstract] OR "implementation science"[MeSH Terms]) AND ("framework*"[Title/Abstract] OR "model*"[Title/Abstract] OR "tool*"[Title/Abstract] OR "implementation framework*"[Title/Abstract] OR "consolidated framework for implementation research"[Title/Abstract] OR "framework synthesis"[Title/Abstract]) AND ("review*"[Title/Abstract] OR "systematic review*"[Title/Abstract] OR "review literature as topic"[MeSH Terms] OR "systematic reviews as topic"[MeSH Terms] OR "meta analysis as topic"[MeSH Terms])) AND ((2012:3000/12/12[pdat]) AND (english[Filter]))  **Translations**  **Review literature as topic[MeSH]:** "review literature as topic"[MeSH Terms]  **Systematic reviews as topic[MeSH]:** "systematic reviews as topic"[MeSH Terms]  **Meta-analysis as topic[MeSH]:** "meta-analysis as topic"[MeSH Terms] | [3,754](https://pubmed.ncbi.nlm.nih.gov/?term=%28%28%22implementation%22%5BTitle%5D+OR+%22determinants+of+implementation%22%5BTitle%2FAbstract%5D+OR+%22implementation+science%22%5BTitle%2FAbstract%5D+OR+%22implementation-effectiveness%22%5BTitle%2FAbstract%5D+OR+%22implementation+evaluation%22%5BTitle%2FAbstract%5D+OR+%22implementation+determinant%2A%22%5BTitle%2FAbstract%5D+OR+%22implementation+outcome%2A%22%5BTitle%2FAbstract%5D+OR+%22implementation+science%22%5BMeSH+Terms%5D%29+AND+%28%28%28%28%28%28framework%2A%5BTitle%2FAbstract%5D%29+OR+%28model%2A%5BTitle%2FAbstract%5D%29%29+OR+%28tool%2A%5BTitle%2FAbstract%5D%29%29+OR+%28implementation+framework%2A%5BTitle%2FAbstract%5D%29%29+OR+%28%22consolidated+framework+for+implementation+research%22%5BTitle%2FAbstract%5D%29%29+OR+%28%22framework+synthesis%22%5BTitle%2FAbstract%5D%29%29%29+AND+%28%28%28%28%28review%2A%5BTitle%2FAbstract%5D%29+OR+%28systematic+review%2A%5BTitle%2FAbstract%5D%29%29+OR+%28Review+literature+as+topic%5BMeSH%5D%29%29+OR+%28Systematic+reviews+as+topic%5BMeSH%5D%29%29+OR+%28Meta-analysis+as+topic%5BMeSH%5D%29%29&filter=dates.2012-3000%2F12%2F12&filter=lang.english&ac=no&show_snippets=off&sort=date&size=200) | 08:20:45 |
| #5 |  |  | Search: **(("implementation"[Title] OR "determinants of implementation"[Title/Abstract] OR "implementation science"[Title/Abstract] OR "implementation-effectiveness"[Title/Abstract] OR "implementation evaluation"[Title/Abstract] OR "implementation determinant*"[Title/Abstract] OR "implementation outcome*"[Title/Abstract] OR "implementation science"[MeSH Terms]) AND ((((((framework*[Title/Abstract]) OR (model*[Title/Abstract])) OR (tool*[Title/Abstract])) OR (implementation framework*[Title/Abstract])) OR ("consolidated framework for implementation research"[Title/Abstract])) OR ("framework synthesis"[Title/Abstract]))) AND (((((review*[Title/Abstract]) OR (systematic review*[Title/Abstract])) OR (Review literature as topic[MeSH])) OR (Systematic reviews as topic[MeSH])) OR (Meta-analysis as topic[MeSH]))** Filters: **from 2012 - 3000/12/12** Sort by: **Most Recent** | [3,806](https://pubmed.ncbi.nlm.nih.gov/?term=%28%28%22implementation%22%5BTitle%5D+OR+%22determinants+of+implementation%22%5BTitle%2FAbstract%5D+OR+%22implementation+science%22%5BTitle%2FAbstract%5D+OR+%22implementation-effectiveness%22%5BTitle%2FAbstract%5D+OR+%22implementation+evaluation%22%5BTitle%2FAbstract%5D+OR+%22implementation+determinant%2A%22%5BTitle%2FAbstract%5D+OR+%22implementation+outcome%2A%22%5BTitle%2FAbstract%5D+OR+%22implementation+science%22%5BMeSH+Terms%5D%29+AND+%28%28%28%28%28%28framework%2A%5BTitle%2FAbstract%5D%29+OR+%28model%2A%5BTitle%2FAbstract%5D%29%29+OR+%28tool%2A%5BTitle%2FAbstract%5D%29%29+OR+%28implementation+framework%2A%5BTitle%2FAbstract%5D%29%29+OR+%28%22consolidated+framework+for+implementation+research%22%5BTitle%2FAbstract%5D%29%29+OR+%28%22framework+synthesis%22%5BTitle%2FAbstract%5D%29%29%29+AND+%28%28%28%28%28review%2A%5BTitle%2FAbstract%5D%29+OR+%28systematic+review%2A%5BTitle%2FAbstract%5D%29%29+OR+%28Review+literature+as+topic%5BMeSH%5D%29%29+OR+%28Systematic+reviews+as+topic%5BMeSH%5D%29%29+OR+%28Meta-analysis+as+topic%5BMeSH%5D%29%29&filter=dates.2012-3000%2F12%2F12&ac=no&show_snippets=off&sort=date&size=200) | 08:20:34 |
| #4 |  |  | Search: **(("implementation"[Title] OR "determinants of implementation"[Title/Abstract] OR "implementation science"[Title/Abstract] OR "implementation-effectiveness"[Title/Abstract] OR "implementation evaluation"[Title/Abstract] OR "implementation determinant*"[Title/Abstract] OR "implementation outcome*"[Title/Abstract] OR "implementation science"[MeSH Terms]) AND ((((((framework*[Title/Abstract]) OR (model*[Title/Abstract])) OR (tool*[Title/Abstract])) OR (implementation framework*[Title/Abstract])) OR ("consolidated framework for implementation research"[Title/Abstract])) OR ("framework synthesis"[Title/Abstract]))) AND (((((review*[Title/Abstract]) OR (systematic review*[Title/Abstract])) OR (Review literature as topic[MeSH])) OR (Systematic reviews as topic[MeSH])) OR (Meta-analysis as topic[MeSH]))** Sort by: **Most Recent** | [4,211](https://pubmed.ncbi.nlm.nih.gov/?term=%28%28%22implementation%22%5BTitle%5D+OR+%22determinants+of+implementation%22%5BTitle%2FAbstract%5D+OR+%22implementation+science%22%5BTitle%2FAbstract%5D+OR+%22implementation-effectiveness%22%5BTitle%2FAbstract%5D+OR+%22implementation+evaluation%22%5BTitle%2FAbstract%5D+OR+%22implementation+determinant%2A%22%5BTitle%2FAbstract%5D+OR+%22implementation+outcome%2A%22%5BTitle%2FAbstract%5D+OR+%22implementation+science%22%5BMeSH+Terms%5D%29+AND+%28%28%28%28%28%28framework%2A%5BTitle%2FAbstract%5D%29+OR+%28model%2A%5BTitle%2FAbstract%5D%29%29+OR+%28tool%2A%5BTitle%2FAbstract%5D%29%29+OR+%28implementation+framework%2A%5BTitle%2FAbstract%5D%29%29+OR+%28%22consolidated+framework+for+implementation+research%22%5BTitle%2FAbstract%5D%29%29+OR+%28%22framework+synthesis%22%5BTitle%2FAbstract%5D%29%29%29+AND+%28%28%28%28%28review%2A%5BTitle%2FAbstract%5D%29+OR+%28systematic+review%2A%5BTitle%2FAbstract%5D%29%29+OR+%28Review+literature+as+topic%5BMeSH%5D%29%29+OR+%28Systematic+reviews+as+topic%5BMeSH%5D%29%29+OR+%28Meta-analysis+as+topic%5BMeSH%5D%29%29&sort=date&size=200&show_snippets=off&ac=no) | 08:20:08 |
| #3 |  |  | Search: **((((review*[Title/Abstract]) OR (systematic review*[Title/Abstract])) OR (Review literature as topic[MeSH])) OR (Systematic reviews as topic[MeSH])) OR (Meta-analysis as topic[MeSH])** Sort by: **Most Recent**  "review*"[Title/Abstract] OR "systematic review*"[Title/Abstract] OR "review literature as topic"[MeSH Terms] OR "systematic reviews as topic"[MeSH Terms] OR "meta analysis as topic"[MeSH Terms]  **Translations**  **Review literature as topic[MeSH]:** "review literature as topic"[MeSH Terms]  **Systematic reviews as topic[MeSH]:** "systematic reviews as topic"[MeSH Terms]  **Meta-analysis as topic[MeSH]:** "meta-analysis as topic"[MeSH Terms] | [2,831,194](https://pubmed.ncbi.nlm.nih.gov/?term=%28%28%28%28review%2A%5BTitle%2FAbstract%5D%29+OR+%28systematic+review%2A%5BTitle%2FAbstract%5D%29%29+OR+%28Review+literature+as+topic%5BMeSH%5D%29%29+OR+%28Systematic+reviews+as+topic%5BMeSH%5D%29%29+OR+%28Meta-analysis+as+topic%5BMeSH%5D%29&sort=date&size=200&show_snippets=off&ac=no) | 08:18:16 |
| #2 |  |  | Search: **(((((framework*[Title/Abstract]) OR (model*[Title/Abstract])) OR (tool*[Title/Abstract])) OR (implementation framework*[Title/Abstract])) OR ("consolidated framework for implementation research"[Title/Abstract])) OR ("framework synthesis"[Title/Abstract])** Sort by: **Most Recent**  "framework*"[Title/Abstract] OR "model*"[Title/Abstract] OR "tool*"[Title/Abstract] OR "implementation framework*"[Title/Abstract] OR "consolidated framework for implementation research"[Title/Abstract] OR "framework synthesis"[Title/Abstract] | [4,891,727](https://pubmed.ncbi.nlm.nih.gov/?term=%28%28%28%28%28framework%2A%5BTitle%2FAbstract%5D%29+OR+%28model%2A%5BTitle%2FAbstract%5D%29%29+OR+%28tool%2A%5BTitle%2FAbstract%5D%29%29+OR+%28implementation+framework%2A%5BTitle%2FAbstract%5D%29%29+OR+%28%22consolidated+framework+for+implementation+research%22%5BTitle%2FAbstract%5D%29%29+OR+%28%22framework+synthesis%22%5BTitle%2FAbstract%5D%29&sort=date&size=200&show_snippets=off&ac=no) | 08:15:50 |
| #1 |  |  | Search: **"implementation"[Title] OR "determinants of implementation"[Title/Abstract] OR "implementation science"[Title/Abstract] OR "implementation-effectiveness"[Title/Abstract] OR "implementation evaluation"[Title/Abstract] OR "implementation determinant*"[Title/Abstract] OR "implementation outcome*"[Title/Abstract] OR "implementation science"[MeSH Terms]** Sort by: **Most Recent**  "implementation"[Title] OR "determinants of implementation"[Title/Abstract] OR "implementation science"[Title/Abstract] OR "implementation-effectiveness"[Title/Abstract] OR "implementation evaluation"[Title/Abstract] OR "implementation determinant*"[Title/Abstract] OR "implementation outcome*"[Title/Abstract] OR "implementation science"[MeSH Terms] | [60,621](https://pubmed.ncbi.nlm.nih.gov/?term=%22implementation%22%5BTitle%5D+OR+%22determinants+of+implementation%22%5BTitle%2FAbstract%5D+OR+%22implementation+science%22%5BTitle%2FAbstract%5D+OR+%22implementation-effectiveness%22%5BTitle%2FAbstract%5D+OR+%22implementation+evaluation%22%5BTitle%2FAbstract%5D+OR+%22implementation+determinant%2A%22%5BTitle%2FAbstract%5D+OR+%22implementation+outcome%2A%22%5BTitle%2FAbstract%5D+OR+%22implementation+science%22%5BMeSH+Terms%5D&sort=date&size=200&show_snippets=off&ac=no) | 08:12:51 |

Search columns:

| Implementation | Framework | Review |
| --- | --- | --- |
| **"implementation"[Title] OR "determinants of implementation"[Title/Abstract] OR "implementation science"[Title/Abstract] OR "implementation-effectiveness"[Title/Abstract] OR "implementation evaluation"[Title/Abstract] OR "implementation determinant*"[Title/Abstract] OR "implementation outcome*"[Title/Abstract] OR "implementation science"[MeSH Terms]** | **(((((framework*[Title/Abstract]) OR (model*[Title/Abstract])) OR (tool*[Title/Abstract])) OR (implementation framework*[Title/Abstract])) OR ("consolidated framework for implementation research"[Title/Abstract])) OR ("framework synthesis"[Title/Abstract])** | **((((review*[Title/Abstract]) OR (systematic review*[Title/Abstract])) OR (Review literature as topic[MeSH])) OR (Systematic reviews as topic[MeSH])) OR (Meta-analysis as topic[MeSH])** |
| **60,621 hits** | **4,891,727 hits** | **2,831,194 hits** |
| Search: **(("implementation"[Title] OR "determinants of implementation"[Title/Abstract] OR "implementation science"[Title/Abstract] OR "implementation-effectiveness"[Title/Abstract] OR "implementation evaluation"[Title/Abstract] OR "implementation determinant*"[Title/Abstract] OR "implementation outcome*"[Title/Abstract] OR "implementation science"[MeSH Terms]) AND ((((((framework*[Title/Abstract]) OR (model*[Title/Abstract])) OR (tool*[Title/Abstract])) OR (implementation framework*[Title/Abstract])) OR ("consolidated framework for implementation research"[Title/Abstract])) OR ("framework synthesis"[Title/Abstract]))) AND (((((review*[Title/Abstract]) OR (systematic review*[Title/Abstract])) OR (Review literature as topic[MeSH])) OR (Systematic reviews as topic[MeSH])) OR (Meta-analysis as topic[MeSH]))** Filters: **English, from 2012 - 3000/12/12** Sort by: **Most Recent**  (("implementation"[Title] OR "determinants of implementation"[Title/Abstract] OR "implementation science"[Title/Abstract] OR "implementation-effectiveness"[Title/Abstract] OR "implementation evaluation"[Title/Abstract] OR "implementation determinant*"[Title/Abstract] OR "implementation outcome*"[Title/Abstract] OR "implementation science"[MeSH Terms]) AND ("framework*"[Title/Abstract] OR "model*"[Title/Abstract] OR "tool*"[Title/Abstract] OR "implementation framework*"[Title/Abstract] OR "consolidated framework for implementation research"[Title/Abstract] OR "framework synthesis"[Title/Abstract]) AND ("review*"[Title/Abstract] OR "systematic review*"[Title/Abstract] OR "review literature as topic"[MeSH Terms] OR "systematic reviews as topic"[MeSH Terms] OR "meta analysis as topic"[MeSH Terms])) AND ((2012:3000/12/12[pdat]) AND (english[Filter]))  **Translations**  **Review literature as topic[MeSH]:** "review literature as topic"[MeSH Terms]  **Systematic reviews as topic[MeSH]:** "systematic reviews as topic"[MeSH Terms]  **Meta-analysis as topic[MeSH]:** "meta-analysis as topic"[MeSH Terms] | | |
| Updated search 27/10/2023 | | |
| Search: **(("implementation"[Title] OR "determinants of implementation"[Title/Abstract] OR "implementation science"[Title/Abstract] OR "implementation-effectiveness"[Title/Abstract] OR "implementation evaluation"[Title/Abstract] OR "implementation determinant*"[Title/Abstract] OR "implementation outcome*"[Title/Abstract] OR "implementation science"[MeSH Terms]) AND ((((((framework*[Title/Abstract]) OR (model*[Title/Abstract])) OR (tool*[Title/Abstract])) OR (implementation framework*[Title/Abstract])) OR ("consolidated framework for implementation research"[Title/Abstract])) OR ("framework synthesis"[Title/Abstract]))) AND (((((review*[Title/Abstract]) OR (systematic review*[Title/Abstract])) OR (Review literature as topic[MeSH])) OR (Systematic reviews as topic[MeSH])) OR (Meta-analysis as topic[MeSH]))** Filters: **English, from 2012 - 3000/12/12**  (("implementation"[Title] OR "determinants of implementation"[Title/Abstract] OR "implementation science"[Title/Abstract] OR "implementation-effectiveness"[Title/Abstract] OR "implementation evaluation"[Title/Abstract] OR "implementation determinant*"[Title/Abstract] OR "implementation outcome*"[Title/Abstract] OR "implementation science"[MeSH Terms]) AND ("framework*"[Title/Abstract] OR "model*"[Title/Abstract] OR "tool*"[Title/Abstract] OR "implementation framework*"[Title/Abstract] OR "consolidated framework for implementation research"[Title/Abstract] OR "framework synthesis"[Title/Abstract]) AND ("review*"[Title/Abstract] OR "systematic review*"[Title/Abstract] OR "review literature as topic"[MeSH Terms] OR "systematic reviews as topic"[MeSH Terms] OR "meta analysis as topic"[MeSH Terms])) AND ((2012:3000/12/12[pdat]) AND (english[Filter]))  **Translations**  **Review literature as topic[MeSH]:** "review literature as topic"[MeSH Terms]  **Systematic reviews as topic[MeSH]:** "systematic reviews as topic"[MeSH Terms]  **Meta-analysis as topic[MeSH]:** "meta-analysis as topic"[MeSH Terms] | | |
| **3,759 hits** | | |

# APA PsycNet

17^th^ January 2025

First attempt using the string and specifying to 2023-2024:

**No matches found for your search:** **Any Field**: (((Any Field: AnyFieldFilt: (Any Field: "Literature Review")) OR (title: ("review*") OR title: ("systematic review*")) OR (abstract: ("review*") OR abstract: ("systematic review*"))) AND ((Year: [2012 TO 9999]))) AND (((title: ("framework*") OR title: ("model*") OR title: ("tool*") OR title: ("implementation framework*") OR title: ("consolidated framework for implementation research") OR title: ("framework synthesis")) OR (abstract: ("framework*") OR abstract: ("model*") OR abstract: ("tool*") OR abstract: ("implementation framework*") OR abstract: ("consolidated framework for implementation research") OR abstract: ("framework synthesis"))) AND ((Year: [2012 TO 9999]))) AND (((title: ("implementation") OR title: ("determinants of implementation") OR title: ("implementation science") OR title: ("implementation-effectiveness") OR title: ("implementation evaluation") OR title: ("implementation determinant*") OR title: ("implementation outcome*")) OR (abstract: ("determinants of implementation") OR abstract: ("implementation science") OR abstract: ("implementation-effectiveness") OR abstract: ("implementation evaluation") OR abstract: ("implementation determinant*") OR abstract: ("implementation outcome*"))) AND ((Year: [2023 TO 2024])))

Second attempt to control for validity of first attempt combining single term search with OR for each block and afterwards combining the three blocks with AND:

**No matches found for your search:** (((**title**: ("implementation")) *OR* (**title**: ("determinants of implementation")) *OR* (**title**: ("implementation science")) *OR* (**title**: ("implementation-effectiveness")) *OR* (**title**: ("implementation evaluation")) *OR* (**title**: ("implementation determinant*")) *OR* (**Any Field**: ("implementation outcome*"))) *OR* ((**abstract**: ("determinants of implementation")) *OR* (**abstract**: ("implementation science")) *OR* (**abstract**: ("implementation-effectiveness")) *OR* (**abstract**: ("implementation evaluation")) *OR* (**abstract**: ("implementation determinant*")) *OR* (**abstract**: ("implementation outcome*"))))) *AND* ((**Year**: [2023 TO 2024] *OR* **TestYear**: [2023 TO 2024]))) *AND* (((**title**: ("framework*")) *OR* (**title**: ("model*")) *OR* (**title**: ("tool*")) *OR* (**title**: ("implementation framework*")) *OR* (**title**: ("consolidated framework for implementation research")) *OR* (**title**: ("framework synthesis"))) *OR* ((**abstract**: ("framework*")) *OR* (**abstract**: ("model*")) *OR* (**abstract**: ("tool*")) *OR* (**abstract**: ("implementation framework*")) *OR* (**abstract**: ("consolidated framework for implementation research")) *OR* (**abstract**: ("framework synthesis"))))) *AND* ((**Year**: [2023 TO 2024] *OR* **TestYear**: [2023 TO 2024]))) *AND* (((AnyField:(AnyFieldFilt:) (AnyField:("Literature Review"))) OR (title:("review*")) OR (title:("systematic review*")) OR (abstract:("review*")) OR (abstract:("systematic review*"))) AND ((PublicationYear:[2023 TO 2024] OR TestYear:[2023 TO 2024])))

Results from the three blocks independently:

**6**Results for **title**: ("implementation") *OR* **title**: ("determinants of implementation") *OR* **title**: ("implementation science") *OR* **title**: ("implementation-effectiveness") *OR* **title**: ("implementation evaluation") *OR* **title**: ("implementation determinant*") *OR* (**Any Field**: "implementation outcome*")) *OR* (**abstract**: ("determinants of implementation") *OR* **abstract**: ("implementation science") *OR* **abstract**: ("implementation-effectiveness") *OR* **abstract**: ("implementation evaluation") *OR* **abstract**: ("implementation determinant*") *OR* **abstract**: ("implementation outcome*"))) *AND* **Year**: 2023 *To* 2024

**1,012**Results for **title**: ("framework*") *OR* **title**: ("model*") *OR* **title**: ("tool*") *OR* **title**: ("implementation framework*") *OR* **title**: ("consolidated framework for implementation research") *OR* **title**: ("framework synthesis")) *OR* (**abstract**: ("framework*") *OR* **abstract**: ("model*") *OR* **abstract**: ("tool*") *OR* **abstract**: ("implementation framework*") *OR* **abstract**: ("consolidated framework for implementation research") *OR* **abstract**: ("framework synthesis"))) *AND* **Year**: 2023 *To* 2024

**605**Results for **Any Field**: AnyFieldFilt: (**Any Field**: "Literature Review") *OR* **title**: ("review*") *OR* **title**: ("systematic review*") *OR* **abstract**: ("review*") *OR* **abstract**: ("systematic review*") *AND* **Year**: 2023 *To* 2024

-------------------------

2023:

| Implementation | Framework | Review |
| --- | --- | --- |
| implementation (Title) OR  determinants of implementation (Title/abstract) OR  implementation science (Title/abstract) OR  implementation-effectiveness (Title/abstract) OR  implementation evaluation (Title/abstract) OR  implementation determinant* (Title/abstract) OR  Implementation outcome* (Title/abstract) ~~OR~~  ~~implementation science (MeSH)~~ | framework* (Title/abstract) OR  model* (Title/abstract) OR  tool* (Title/abstract) OR  implementation framework* (Title/abstract) OR  consolidated framework for implementation research (Title/abstract) OR  framework synthesis (Title/abstract) | review* (Title/abstract) OR  systematic review* (Title/abstract) OR  {Literature Review} ~~OR~~  ~~Review literature as topic (MeSH) OR~~  ~~Systematic reviews as topic (MeSH) OR~~  ~~Meta-analysis as topic (MeSH)~~ |
| PsycNet search | | |
| (((Title:("implementation") OR Title:("determinants of implementation") OR Title:("implementation science") OR Title:("implementation-effectiveness") OR Title:("implementation evaluation") OR Title:("implementation determinant*") OR Title:("implementation outcome*")) OR (Abstract:("determinants of implementation") OR Abstract:("implementation science") OR Abstract:("implementation-effectiveness") OR Abstract:("implementation evaluation") OR Abstract:("implementation determinant*") OR Abstract:("implementation outcome*"))) AND ((PublicationYear:[2012 TO 9999]))) | (((Title:("framework*") OR Title:("model*") OR Title:("tool*") OR Title:("implementation framework*") OR Title:("consolidated framework for implementation research") OR Title:("framework synthesis")) OR (Abstract:("framework*") OR Abstract:("model*") OR Abstract:("tool*") OR Abstract:("implementation framework*") OR Abstract:("consolidated framework for implementation research") OR Abstract:("framework synthesis"))) AND ((PublicationYear:[2012 TO 9999]))) | (((AnyFieldFilt:("Literature Review")) OR (Title:("review*") OR Title:("systematic review*")) OR (Abstract:("review*") OR Abstract:("systematic review*"))) AND ((PublicationYear:[2012 TO 9999]))) |
| 12,044 hits | 653,365 hits | 252,119 hits |
| **898**Results for (((**Any Field**: AnyFieldFilt: (**Any Field**: "Literature Review")) *OR* (**title**: ("review*") *OR* **title**: ("systematic review*")) *OR* (**abstract**: ("review*") *OR* **abstract**: ("systematic review*"))) *AND* ((**Year**: [2012 TO 9999]))) *AND* (((**title**: ("framework*") *OR* **title**: ("model*") *OR* **title**: ("tool*") *OR* **title**: ("implementation framework*") *OR* **title**: ("consolidated framework for implementation research") *OR* **title**: ("framework synthesis")) *OR* (**abstract**: ("framework*") *OR* **abstract**: ("model*") *OR* **abstract**: ("tool*") *OR* **abstract**: ("implementation framework*") *OR* **abstract**: ("consolidated framework for implementation research") *OR* **abstract**: ("framework synthesis"))) *AND* ((**Year**: [2012 TO 9999]))) *AND* (((**title**: ("implementation") *OR* **title**: ("determinants of implementation") *OR* **title**: ("implementation science") *OR* **title**: ("implementation-effectiveness") *OR* **title**: ("implementation evaluation") *OR* **title**: ("implementation determinant*") *OR* **title**: ("implementation outcome*")) *OR* (**abstract**: ("determinants of implementation") *OR* **abstract**: ("implementation science") *OR* **abstract**: ("implementation-effectiveness") *OR* **abstract**: ("implementation evaluation") *OR* **abstract**: ("implementation determinant*") *OR* **abstract**: ("implementation outcome*"))) *AND* ((**Year**: [2012 TO 9999]))) | | |
| **898 hits** | | |

Only one thesaurus term available in Psychinfo, i.e., {Literature Review}.

Search string from PubMed was copied to PsycINFO/PsycNet, without the Title/Abstract specification. As it is not possible to search both Title and Abstract at once, two search string (one with Title specification and one with Abstract specification were combined with the Boolean operator OR). When all search terms of the column were included in the Advanced Search format, the Search was performed (see screenshot in figure 1).


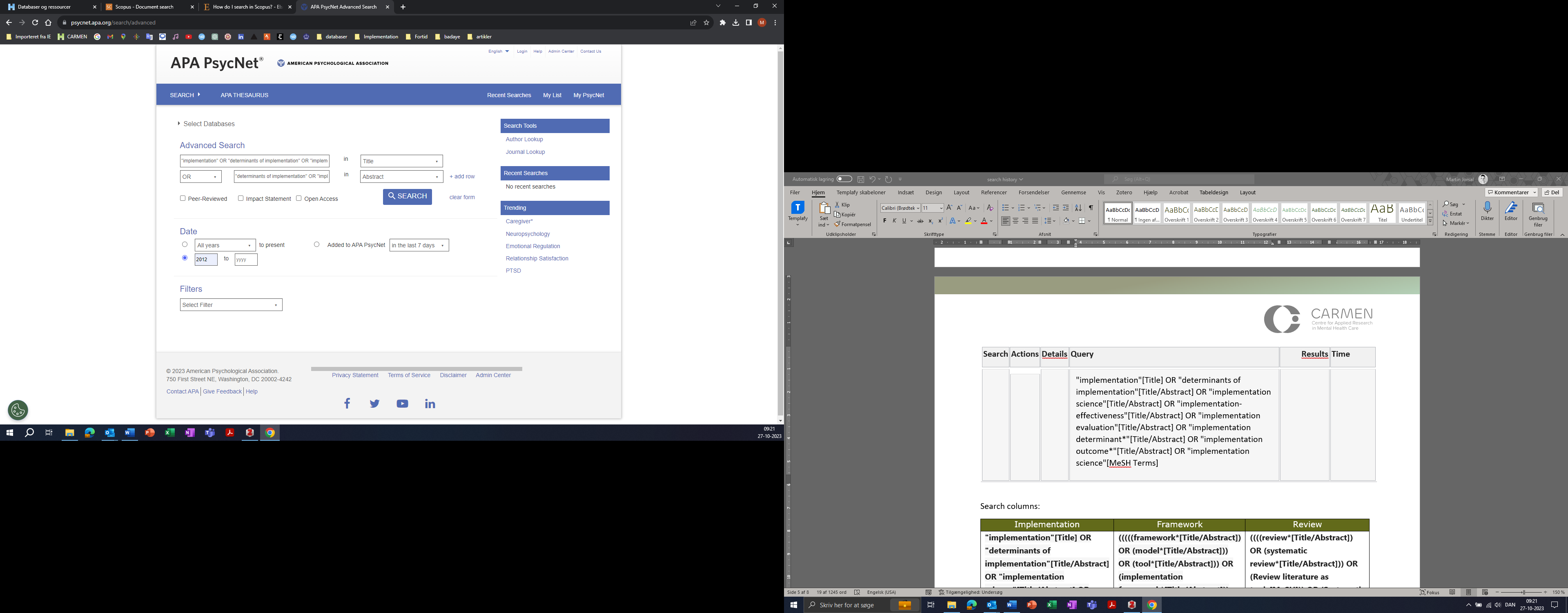


Figure 1 - search column "implementation"

When search of all three columns of the search matrix had been performed, the columns were combined with Boolean operator AND (see screenshot In figure 2).


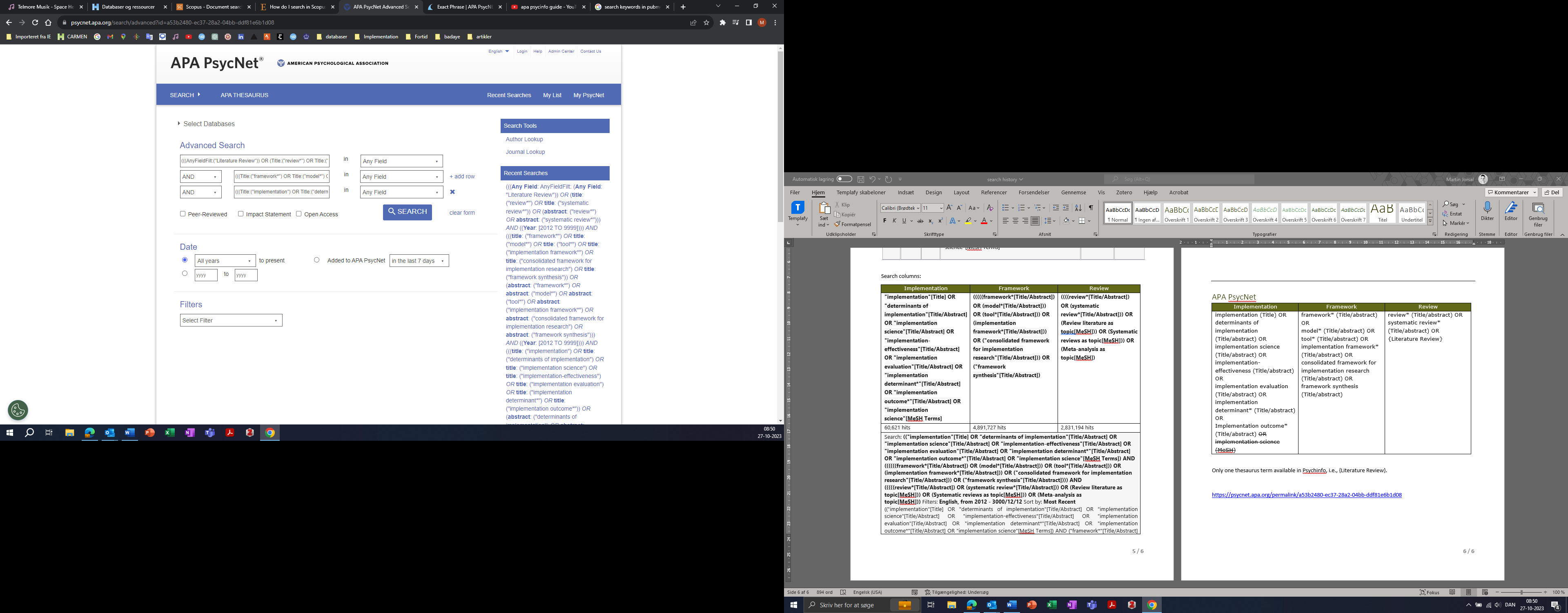


Figure 2 - full search

The full search string was as follows:

**898**Results for (((**Any Field**: AnyFieldFilt: (**Any Field**: "Literature Review")) *OR* (**title**: ("review*") *OR* **title**: ("systematic review*")) *OR* (**abstract**: ("review*") *OR* **abstract**: ("systematic review*"))) *AND* ((**Year**: [2012 TO 9999]))) *AND* (((**title**: ("framework*") *OR* **title**: ("model*") *OR* **title**: ("tool*") *OR* **title**: ("implementation framework*") *OR* **title**: ("consolidated framework for implementation research") *OR* **title**: ("framework synthesis")) *OR* (**abstract**: ("framework*") *OR* **abstract**: ("model*") *OR* **abstract**: ("tool*") *OR* **abstract**: ("implementation framework*") *OR* **abstract**: ("consolidated framework for implementation research") *OR* **abstract**: ("framework synthesis"))) *AND* ((**Year**: [2012 TO 9999]))) *AND* (((**title**: ("implementation") *OR* **title**: ("determinants of implementation") *OR* **title**: ("implementation science") *OR* **title**: ("implementation-effectiveness") *OR* **title**: ("implementation evaluation") *OR* **title**: ("implementation determinant*") *OR* **title**: ("implementation outcome*")) *OR* (**abstract**: ("determinants of implementation") *OR* **abstract**: ("implementation science") *OR* **abstract**: ("implementation-effectiveness") *OR* **abstract**: ("implementation evaluation") *OR* **abstract**: ("implementation determinant*") *OR* **abstract**: ("implementation outcome*"))) *AND* ((**Year**: [2012 TO 9999])))

<https://psycnet.apa.org/permalink/a53b2480-ec37-28a2-04bb-ddf81e6b1d08>

Search results were downloaded and exported to Zotero.

# Scopus

17^th^ January 2025

*Troubles specifiying for October 2023 – December 2024. Tried with syntax PUBDATETXT("2023-10-01" AND "2024-12-31") with no positive effect as it led to 0 results. Could only specify to publication year, and not publication date. Further specification must be done manually.*

( ( TITLE ( "implementation" ) OR TITLE-ABS-KEY ( "determinants of implementation" OR "implementation science" OR "implementation-effectiveness" OR "implementation evaluation" OR "implementation determinant*" OR "implementation outcome*" ) ) AND PUBYEAR > 2011 ) AND ( TITLE-ABS-KEY ( "framework*" OR "model*" OR "tool*" OR "implementation framework*" OR "consolidated framework for implementation research" OR "framework synthesis" ) AND PUBYEAR > 2011 ) AND ( TITLE-ABS-KEY ( "review*" OR "systematic review*" ) AND PUBYEAR > 2011 ) AND PUBYEAR > 2022 AND PUBYEAR < 2025 AND ( LIMIT-TO ( LANGUAGE , "English" ) ) AND ( LIMIT-TO ( DOCTYPE , "re" ) ) AND ( LIMIT-TO ( SUBJAREA , "MEDI" ) )

574 hits

------

2023

| Implementation | Framework | Review |
| --- | --- | --- |
| implementation (Title) OR  determinants of implementation (Title/abstract) OR  implementation science (Title/abstract) OR  implementation-effectiveness (Title/abstract) OR  implementation evaluation (Title/abstract) OR  implementation determinant* (Title/abstract) OR  Implementation outcome* (Title/abstract) ~~OR~~  ~~implementation science (MeSH)~~ | framework* (Title/abstract) OR  model* (Title/abstract) OR  tool* (Title/abstract) OR  implementation framework* (Title/abstract) OR  consolidated framework for implementation research (Title/abstract) OR  framework synthesis (Title/abstract) | review* (Title/abstract) OR  systematic review* (Title/abstract)  ~~OR~~  ~~Review literature as topic (MeSH) OR~~  ~~Systematic reviews as topic (MeSH) OR~~  ~~Meta-analysis as topic (MeSH)~~ |
| Scopus search | | |
| ( TITLE ( "implementation" ) OR TITLE-ABS-KEY ( "determinants of implementation" OR "implementation science" OR "implementation-effectiveness" OR "implementation evaluation" OR "implementation determinant*" OR "implementation outcome*" ) ) AND PUBYEAR > 2011 AND ( LIMIT-TO ( LANGUAGE , "English" ) ) | TITLE-ABS-KEY ( "framework*" OR "model*" OR "tool*" OR "implementation framework*" OR "consolidated framework for implementation research" OR "framework synthesis" ) AND PUBYEAR > 2011 AND ( LIMIT-TO ( LANGUAGE , "English" ) ) | TITLE-ABS-KEY ( "review*" OR "systematic review*" ) AND PUBYEAR > 2011 AND ( LIMIT-TO ( LANGUAGE , "English" ) ) |
| 170,189 hits | 10,911,765 hits | 3,187,825 hits |
| ( ( TITLE ( "implementation" ) OR TITLE-ABS-KEY ( "determinants of implementation" OR "implementation science" OR "implementation-effectiveness" OR "implementation evaluation" OR "implementation determinant*" OR "implementation outcome*" ) ) AND PUBYEAR > 2011 ) AND ( TITLE-ABS-KEY ( "framework*" OR "model*" OR "tool*" OR "implementation framework*" OR "consolidated framework for implementation research" OR "framework synthesis" ) AND PUBYEAR > 2011 ) AND ( TITLE-ABS-KEY ( "review*" OR "systematic review*" ) AND PUBYEAR > 2011 ) AND ( LIMIT-TO ( LANGUAGE , "English" ) ) | | |
| **7,751 hits** | | |

The search method on Scopus follows the same procedure as in PsycNet, but with minor differences.

Scopus does not imply Thesaurus (such as MeSH terms in PubMed), which was therefore excluded in this search.

## New Scopus search on 6^th^ of November 2023

After guidance from Georgia Fisher from Centre for Healthcare Resilience and Implementation Science at MacQuirie University, Australia, a new search was performed in the Scopus database on the November 6, 2023.

Two search strings were searched in advanced search with filters on English language, year 2012< and review applied. Search string were as follows:

TITLE-ABS-KEY ( "quality implementation framework" OR "implementation model*" OR "implementation framework*" OR "implementation tool*" OR "determinants of implementation" OR "implementation science" OR "implementation effectiveness" OR "implementation evaluation*" OR "implementation determinant*" OR "implementation outcome*" OR "implementation science model*" OR "implementation science framework*" OR "implementation science tool*" ) AND TITLE-ABS-KEY ( "review" ) AND PUBYEAR > 2010 AND PUBYEAR < 2025 AND PUBYEAR > 2011 AND PUBYEAR < 2024 AND ( LIMIT-TO ( DOCTYPE , "re" ) ) AND ( LIMIT-TO ( LANGUAGE , "english" ) ) **1,451 hits**

TITLE-ABS-KEY ( "quality implementation framework" OR ( "implementation" W/2 "model*" ) OR ( "implementation" W/2 "framework*" ) OR ( "implementation*" W/2 "tool*" ) OR "implementation evaluation" OR "determinants of implementation" OR "implementation science" OR "implementation-effectiveness" OR "implementation evaluation" OR "implementation determinant*" OR "implementation outcome*" OR ( "implementation science" W/2 "model*" ) OR ( "implementation science" W/2 "framework*" ) OR ( "implementation* science" W/2 "tool*" ) ) AND TITLE-ABS-KEY ( "review" ) AND PUBYEAR > 2010 AND PUBYEAR < 2025 AND PUBYEAR > 2011 AND PUBYEAR < 2025 AND ( LIMIT-TO ( DOCTYPE , "re" ) ) AND ( LIMIT-TO ( LANGUAGE , "english" ) ) **2,514 hits**

**The largest (i.e., 2^nd^) search has been imported to Covidence November 6, 2023.**

# Web of Science

22^nd^ January 2025:

Using the 2023 query link and adding filter for 2023-2024.

<https://www.webofscience.com/wos/woscc/summary/2ddbc7b4-dd0b-4f9a-b2b1-4e7c02ae9467-01452e7559/relevance/1>

2023:

| Implementation | Framework | Review |
| --- | --- | --- |
| implementation (Title) OR  determinants of implementation (Title/abstract) OR  implementation science (Title/abstract) OR  implementation-effectiveness (Title/abstract) OR  implementation evaluation (Title/abstract) OR  implementation determinant* (Title/abstract) OR  Implementation outcome* (Title/abstract) ~~OR~~  ~~implementation science (MeSH)~~ | framework* (Title/abstract) OR  model* (Title/abstract) OR  tool* (Title/abstract) OR  implementation framework* (Title/abstract) OR  consolidated framework for implementation research (Title/abstract) OR  framework synthesis (Title/abstract) | review* (Title/abstract) OR  systematic review* (Title/abstract)  ~~OR~~  ~~Review literature as topic (MeSH) OR~~  ~~Systematic reviews as topic (MeSH) OR~~  ~~Meta-analysis as topic (MeSH)~~ |
| Web of Science search | | |
| (TI=("implementation" OR "determinants of implementation" OR "implementation science" OR "implementation-effectiveness" OR "implementation evaluation" OR "implementation determinant*" OR "implementation outcome*" )) OR AB=("determinants of implementation" OR "implementation science" OR "implementation-effectiveness" OR "implementation evaluation" OR "implementation determinant*" OR "implementation outcome*") | (TI=("framework*" OR "model*" OR "tool*" OR "implementation framework*" OR "consolidated framework for implementation research" OR "framework synthesis")) OR AB=("framework*" OR "model*" OR "tool*" OR "implementation framework*" OR "consolidated framework for implementation research" OR "framework synthesis") | (TI=("review*" OR "systematic review*")) AND AB=("review*" OR "systematic review*") |
| 219,175 hits | 13,629,758 hits | 596,089 hits |
| (#1 AND #2 AND #3) AND (LA==("ENGLISH")) | | |
| **1,328 hits** | | |

Query link: <https://www.webofscience.com/wos/woscc/summary/7daedd95-2c52-48a2-8c8d-436a00d6a0ac-aef3e493/relevance/1>

Search method followed same procedure as in Scopus (i.e., no thesaurus was implied). Although not visible in the table above, publication date range was applied as a filter when the three search columns were combined with Boolean operator AND.
