# Supplementary material for: Updating the critical steps of the quality implementation framework: an umbrella review of reviews
Source: Implement Sci Commun. 2026 May 27;7:112. doi: 10.1186/s43058-026-00954-7 (PMC13242111; doi:10.1186/s43058-026-00954-7)
Supplement: Supplementary file 4 — Supplementary Material 4 [file 43058_2026_954_MOESM4_ESM.pdf]

JBI CRITICAL APPRAISAL CHECKLIST FOR SYSTEMATIC REVIEWS AND RESEARCH SYNTHESES

Reviewer KH & AGO Date MAY 2025

Author ALBERS Year 2017 Record Number________

		Yes	No	Unclear	Not applicable
1.	Is the review question clearly and explicitly stated?	☐	☒	☐	☐
2.	Were the inclusion criteria appropriate for the review question?	☒	☐	☐	☐
3.	Was the search strategy appropriate?	☒	☐	☐	☐
4.	Were the sources and resources used to search for studies adequate?	☒	☐	☐	☐
5.	Were the criteria for appraising studies appropriate?	☒	☐	☐	☐
6.	Was critical appraisal conducted by two or more reviewers independently?	☒	☐	☐	☐
7.	Were there methods to minimize errors in data extraction?	☒	☐	☐	☐
8.	Were the methods used to combine studies appropriate?	☒	☐	☐	☐
9.	Was the likelihood of publication bias assessed?	☐	☒	☐	☐
10.	Were recommendations for policy and/or practice supported by the reported data?	☒	☐	☐	☐
11.	Were the specific directives for new research appropriate?	☒	☐	☐	☐

Overall appraisal: Include ☒ Exclude ☐ Seek further info ☐

Comments (Including reason for exclusion)

____

____

____

JBI CRITICAL APPRAISAL CHECKLIST FOR SYSTEMATIC REVIEWS AND RESEARCH SYNTHESES

Reviewer KH & AGO Date MAY 2025

Author CHAUDOIR Year 2013 Record Number________

	Yes	No	Unclear	Not applicable
1. Is the review question clearly and explicitly stated?	☒	☐	☐	☐
2. Were the inclusion criteria appropriate for the review question?	☒	☐	☐	☐
3. Was the search strategy appropriate?	☒	☐	☐	☐
4. Were the sources and resources used to search for studies adequate?	☒	☐	☐	☐
5. Were the criteria for appraising studies appropriate?	☒	☐	☐	☐
6. Was critical appraisal conducted by two or more reviewers independently?	☐	☒	☐	☐
7. Were there methods to minimize errors in data extraction?	☒	☐	☐	☐
8. Were the methods used to combine studies appropriate?	☒	☐	☐	☐
9. Was the likelihood of publication bias assessed?	☐	☒	☐	☐
10. Were recommendations for policy and/or practice supported by the reported data?	☒	☐	☐	☐
11. Were the specific directives for new research appropriate?	☒	☐	☐	☐

Overall appraisal: Include ☒ Exclude ☐ Seek further info ☐

Comments (Including reason for exclusion)

____

____

____

JBI CRITICAL APPRAISAL CHECKLIST FOR SYSTEMATIC REVIEWS AND RESEARCH SYNTHESES

Reviewer: KH & AGO   Date: MAY 2025

Author: ESCOFFERY   Year: 2023   Record Number: ______

	Yes	No	Unclear	Not applicable
1. Is the review question clearly and explicitly stated?	☒	☐	☐	☐
2. Were the inclusion criteria appropriate for the review question?	☐	☐	☒	☐
3. Was the search strategy appropriate?	☐	☐	☒	☐
4. Were the sources and resources used to search for studies adequate?	☐	☐	☒	☐
5. Were the criteria for appraising studies appropriate?	☐	☐	☒	☐
6. Was critical appraisal conducted by two or more reviewers independently?	☐	☒	☐	☐
7. Were there methods to minimize errors in data extraction?	☐	☐	☒	☐
8. Were the methods used to combine studies appropriate?	☒	☐	☐	☐
9. Was the likelihood of publication bias assessed?	☐	☒	☐	☐
10. Were recommendations for policy and/or practice supported by the reported data?	☒	☐	☐	☐
11. Were the specific directives for new research appropriate?	☒	☐	☐	☐

Overall appraisal: Include ☒ Exclude ☐ Seek further info ☐

Comments (Including reason for exclusion)

INCLUDED DESPITE RECEIVING A LOW QUALITY APPRAISAL RATING DUE TO ITS VERY RELEVANT PERSPECTIVES ON OUTER SETTING IN THE IMPLEMENTATION PROCESS.

JBI CRITICAL APPRAISAL CHECKLIST FOR SYSTEMATIC REVIEWS AND RESEARCH SYNTHESES

Reviewer KH & AGO Date MAY 2025

Author HUYBRECHTS Year 2021 Record Number ________

	Yes	No	Unclear	Not applicable
1. Is the review question clearly and explicitly stated?	☒	☐	☐	☐
2. Were the inclusion criteria appropriate for the review question?	☒	☐	☐	☐
3. Was the search strategy appropriate?	☒	☐	☐	☐
4. Were the sources and resources used to search for studies adequate?	☒	☐	☐	☐
5. Were the criteria for appraising studies appropriate?	☒	☐	☐	☐
6. Was critical appraisal conducted by two or more reviewers independently?	☐	☒	☐	☐
7. Were there methods to minimize errors in data extraction?	☒	☐	☐	☐
8. Were the methods used to combine studies appropriate?	☒	☐	☐	☐
9. Was the likelihood of publication bias assessed?	☐	☒	☐	☐
10. Were recommendations for policy and/or practice supported by the reported data?	☒	☐	☐	☐
11. Were the specific directives for new research appropriate?	☒	☐	☐	☐

Overall appraisal: Include ☒ Exclude ☐ Seek further info ☐

Comments (Including reason for exclusion)

____

____

____

JBI CRITICAL APPRAISAL CHECKLIST FOR SYSTEMATIC REVIEWS AND RESEARCH SYNTHESES

Reviewer KH & AGO Date MAY 2025

Author Li Year 2018 Record Number________

	Yes	No	Unclear	Not applicable
1. Is the review question clearly and explicitly stated?	☒	☐	☐	☐
2. Were the inclusion criteria appropriate for the review question?	☒	☐	☐	☐
3. Was the search strategy appropriate?	☒	☐	☐	☐
4. Were the sources and resources used to search for studies adequate?	☒	☐	☐	☐
5. Were the criteria for appraising studies appropriate?	☒	☐	☐	☐
6. Was critical appraisal conducted by two or more reviewers independently?	☒	☐	☐	☐
7. Were there methods to minimize errors in data extraction?	☒	☐	☐	☐
8. Were the methods used to combine studies appropriate?	☒	☐	☐	☐
9. Was the likelihood of publication bias assessed?	☐	☒	☐	☐
10. Were recommendations for policy and/or practice supported by the reported data?	☒	☐	☐	☐
11. Were the specific directives for new research appropriate?	☒	☐	☐	☐

Overall appraisal: Include ☒ Exclude ☐ Seek further info ☐

Comments (Including reason for exclusion)

____

____

____

JBI CRITICAL APPRAISAL CHECKLIST FOR SYSTEMATIC REVIEWS AND RESEARCH SYNTHESES

Reviewer: KH & AGO Date: MAY 2025

Author: MAHMOUD Year: 2023 Record Number: ________

		Yes	No	Unclear	Not applicable
1.	Is the review question clearly and explicitly stated?	☐	☒	☐	☐
2.	Were the inclusion criteria appropriate for the review question?	☐	☐	☒	☐
3.	Was the search strategy appropriate?	☐	☐	☒	☐
4.	Were the sources and resources used to search for studies adequate?	☐	☐	☒	☐
5.	Were the criteria for appraising studies appropriate?	☐	☐	☒	☐
6.	Was critical appraisal conducted by two or more reviewers independently?	☐	☐	☒	☐
7.	Were there methods to minimize errors in data extraction?	☐	☐	☒	☐
8.	Were the methods used to combine studies appropriate?	☒	☐	☐	☐
9.	Was the likelihood of publication bias assessed?	☐	☒	☐	☐
10.	Were recommendations for policy and/or practice supported by the reported data?	☒	☐	☐	☐
11.	Were the specific directives for new research appropriate?	☒	☐	☐	☐

Overall appraisal: Include ☒ Exclude ☐ Seek further info ☐

Comments (Including reason for exclusion)

INCLUDED DESPITE RECEIVING A LOW QUALITY APPAISAL RATING, DUE TO ITS DEVELOPMENT OF A HIGHLY APPLICABLE AND VALUABLE FRAMEWORK!

JBI CRITICAL APPRAISAL CHECKLIST FOR SYSTEMATIC REVIEWS AND RESEARCH SYNTHESES

Reviewer: KH & AGO Date: MAY 2025

Author: McHUGH Year: 2020 Record Number: ______

		Yes	No	Unclear	Not applicable
1.	Is the review question clearly and explicitly stated?	☐	☒	☐	☐
2.	Were the inclusion criteria appropriate for the review question?	☒	☐	☐	☐
3.	Was the search strategy appropriate?	☒	☐	☐	☐
4.	Were the sources and resources used to search for studies adequate?	☒	☐	☐	☐
5.	Were the criteria for appraising studies appropriate?	☒	☐	☐	☐
6.	Was critical appraisal conducted by two or more reviewers independently?	☐	☒	☐	☐
7.	Were there methods to minimize errors in data extraction?	☒	☐	☐	☐
8.	Were the methods used to combine studies appropriate?	☒	☐	☐	☐
9.	Was the likelihood of publication bias assessed?	☐	☒	☐	☐
10.	Were recommendations for policy and/or practice supported by the reported data?	☒	☐	☐	☐
11.	Were the specific directives for new research appropriate?	☒	☐	☐	☐

Overall appraisal: Include ☒ Exclude ☐ Seek further info ☐

Comments (Including reason for exclusion)

JBI CRITICAL APPRAISAL CHECKLIST FOR SYSTEMATIC REVIEWS AND RESEARCH SYNTHESES

Reviewer KH & SPJ Date February 2026

Author Nair Year 2025 Record Number ________

		Yes	No	Unclear	Not applicable
1.	Is the review question clearly and explicitly stated?	☐	☒	☐	☐
2.	Were the inclusion criteria appropriate for the review question?	☐	☐	☒	☐
3.	Was the search strategy appropriate?	☒	☐	☐	☐
4.	Were the sources and resources used to search for studies adequate?	☒	☐	☐	☐
5.	Were the criteria for appraising studies appropriate?	☒	☐	☐	☐
6.	Was critical appraisal conducted by two or more reviewers independently?	☒	☐	☐	☐
7.	Were there methods to minimize errors in data extraction?	☒	☐	☐	☐
8.	Were the methods used to combine studies appropriate?	☒	☐	☐	☐
9.	Was the likelihood of publication bias assessed?	☐	☒	☐	☐
10.	Were recommendations for policy and/or practice supported by the reported data?	☒	☐	☐	☐
11.	Were the specific directives for new research appropriate?	☒	☐	☐	☐

Overall appraisal: Include ☒ Exclude ☐ Seek further info ☐

Comments (Including reason for exclusion)

____

____

____

JBI CRITICAL APPRAISAL CHECKLIST FOR SYSTEMATIC REVIEWS AND RESEARCH SYNTHESES

Reviewer KH & AGO Date MAY 2025

Author NILSEN Year 2019 Record Number________

		Yes	No	Unclear	Not applicable
1.	Is the review question clearly and explicitly stated?	☒	☐	☐	☐
2.	Were the inclusion criteria appropriate for the review question?	☒	☐	☐	☐
3.	Was the search strategy appropriate?	☒	☐	☐	☐
4.	Were the sources and resources used to search for studies adequate?	☒	☐	☐	☐
5.	Were the criteria for appraising studies appropriate?	☒	☐	☐	☐
6.	Was critical appraisal conducted by two or more reviewers independently?	☒	☐	☐	☐
7.	Were there methods to minimize errors in data extraction?	☒	☐	☐	☐
8.	Were the methods used to combine studies appropriate?	☒	☐	☐	☐
9.	Was the likelihood of publication bias assessed?	☐	☒	☐	☐
10.	Were recommendations for policy and/or practice supported by the reported data?	☒	☐	☐	☐
11.	Were the specific directives for new research appropriate?	☒	☐	☐	☐

Overall appraisal: Include ☒ Exclude ☐ Seek further info ☐

Comments (Including reason for exclusion)

____

____

____

JBI CRITICAL APPRAISAL CHECKLIST FOR SYSTEMATIC REVIEWS AND RESEARCH SYNTHESES

Reviewer KH & AGO Date MAY 2025

Author OSTERMEIER Year 2023 Record Number ________

	Yes	No	Unclear	Not applicable
1. Is the review question clearly and explicitly stated?	☒	☐	☐	☐
2. Were the inclusion criteria appropriate for the review question?	☒	☐	☐	☐
3. Was the search strategy appropriate?	☒	☐	☐	☐
4. Were the sources and resources used to search for studies adequate?	☒	☐	☐	☐
5. Were the criteria for appraising studies appropriate?	☒	☐	☐	☐
6. Was critical appraisal conducted by two or more reviewers independently?	☒	☐	☐	☐
7. Were there methods to minimize errors in data extraction?	☒	☐	☐	☐
8. Were the methods used to combine studies appropriate?	☒	☐	☐	☐
9. Was the likelihood of publication bias assessed?	☐	☒	☐	☐
10. Were recommendations for policy and/or practice supported by the reported data?	☒	☐	☐	☐
11. Were the specific directives for new research appropriate?	☒	☐	☐	☐

Overall appraisal: Include ☒ Exclude ☐ Seek further info ☐

Comments (Including reason for exclusion)

JBI CRITICAL APPRAISAL CHECKLIST FOR SYSTEMATIC REVIEWS AND RESEARCH SYNTHESES

Reviewer: KH & AGO Date: MAY 2025

Author: PiAT Year: 2021 Record Number: ______

		Yes	No	Unclear	Not applicable
1.	Is the review question clearly and explicitly stated?	☒	☐	☐	☐
2.	Were the inclusion criteria appropriate for the review question?	☒	☐	☐	☐
3.	Was the search strategy appropriate?	☒	☐	☐	☐
4.	Were the sources and resources used to search for studies adequate?	☒	☐	☐	☐
5.	Were the criteria for appraising studies appropriate?	☒	☐	☐	☐
6.	Was critical appraisal conducted by two or more reviewers independently?	☒	☐	☐	☐
7.	Were there methods to minimize errors in data extraction?	☒	☐	☐	☐
8.	Were the methods used to combine studies appropriate?	☒	☐	☐	☐
9.	Was the likelihood of publication bias assessed?	☐	☒	☐	☐
10.	Were recommendations for policy and/or practice supported by the reported data?	☒	☐	☐	☐
11.	Were the specific directives for new research appropriate?	☒	☐	☐	☐

Overall appraisal: Include ☒ Exclude ☐ Seek further info ☐

Comments (Including reason for exclusion)

____

____

____

JBI CRITICAL APPRAISAL CHECKLIST FOR SYSTEMATIC REVIEWS AND RESEARCH SYNTHESES

Reviewer KH & AGO Date MAY 2025

Author RICHTER Year 2022 Record Number ________

		Yes	No	Unclear	Not applicable
1.	Is the review question clearly and explicitly stated?	☒	☐	☐	☐
2.	Were the inclusion criteria appropriate for the review question?	☒	☐	☐	☐
3.	Was the search strategy appropriate?	☒	☐	☐	☐
4.	Were the sources and resources used to search for studies adequate?	☒	☐	☐	☐
5.	Were the criteria for appraising studies appropriate?	☒	☐	☐	☐
6.	Was critical appraisal conducted by two or more reviewers independently?	☒	☐	☐	☐
7.	Were there methods to minimize errors in data extraction?	☒	☐	☐	☐
8.	Were the methods used to combine studies appropriate?	☒	☐	☐	☐
9.	Was the likelihood of publication bias assessed?	☐	☒	☐	☐
10.	Were recommendations for policy and/or practice supported by the reported data?	☒	☐	☐	☐
11.	Were the specific directives for new research appropriate?	☒	☐	☐	☐

Overall appraisal: Include ☒ Exclude ☐ Seek further info ☐

Comments (Including reason for exclusion)

____

JBI CRITICAL APPRAISAL CHECKLIST FOR SYSTEMATIC REVIEWS AND RESEARCH SYNTHESES

Reviewer KH & SPJ Date February 2026

Author Shierk Year 2025 Record Number________

	Yes	No	Unclear	Not applicable
1. Is the review question clearly and explicitly stated?	☐	☒	☐	☐
2. Were the inclusion criteria appropriate for the review question?	☒	☐	☐	☐
3. Was the search strategy appropriate?	☒	☐	☐	☐
4. Were the sources and resources used to search for studies adequate?	☒	☐	☐	☐
5. Were the criteria for appraising studies appropriate?	☒	☐	☐	☐
6. Was critical appraisal conducted by two or more reviewers independently?	☒	☐	☐	☐
7. Were there methods to minimize errors in data extraction?	☒	☐	☐	☐
8. Were the methods used to combine studies appropriate?	☒	☐	☐	☐
9. Was the likelihood of publication bias assessed?	☐	☒	☐	☐
10. Were recommendations for policy and/or practice supported by the reported data?	☒	☐	☐	☐
11. Were the specific directives for new research appropriate?	☒	☐	☐	☐

Overall appraisal: Include ☒ Exclude ☐ Seek further info ☐

Comments (Including reason for exclusion)

____

____

____

JBI CRITICAL APPRAISAL CHECKLIST FOR SYSTEMATIC REVIEWS AND RESEARCH SYNTHESES

Reviewer: KH & AGO   Date: MAY 2025

Author: TOMS   Year: 2019   Record Number: ________

		Yes	No	Unclear	Not applicable
1.	Is the review question clearly and explicitly stated?	☐	☒	☐	☐
2.	Were the inclusion criteria appropriate for the review question?	☒	☐	☐	☐
3.	Was the search strategy appropriate?	☒	☐	☐	☐
4.	Were the sources and resources used to search for studies adequate?	☒	☐	☐	☐
5.	Were the criteria for appraising studies appropriate?	☒	☐	☐	☐
6.	Was critical appraisal conducted by two or more reviewers independently?	☐	☒	☐	☐
7.	Were there methods to minimize errors in data extraction?	☒	☐	☐	☐
8.	Were the methods used to combine studies appropriate?	☒	☐	☐	☐
9.	Was the likelihood of publication bias assessed?	☐	☒	☐	☐
10.	Were recommendations for policy and/or practice supported by the reported data?	☒	☐	☐	☐
11.	Were the specific directives for new research appropriate?	☒	☐	☐	☐

Overall appraisal: Include ☒ Exclude ☐ Seek further info ☐

Comments (Including reason for exclusion)

____

____

____

JBI CRITICAL APPRAISAL CHECKLIST FOR SYSTEMATIC REVIEWS AND RESEARCH SYNTHESES

Reviewer KH & AGO Date MAY 2025

Author VARSI Year 2019 Record Number________

	Yes	No	Unclear	Not applicable
1. Is the review question clearly and explicitly stated?	☐	☒	☐	☐
2. Were the inclusion criteria appropriate for the review question?	☒	☐	☐	☐
3. Was the search strategy appropriate?	☒	☐	☐	☐
4. Were the sources and resources used to search for studies adequate?	☒	☐	☐	☐
5. Were the criteria for appraising studies appropriate?	☒	☐	☐	☐
6. Was critical appraisal conducted by two or more reviewers independently?	☒	☐	☐	☐
7. Were there methods to minimize errors in data extraction?	☒	☐	☐	☐
8. Were the methods used to combine studies appropriate?	☒	☐	☐	☐
9. Was the likelihood of publication bias assessed?	☐	☒	☐	☐
10. Were recommendations for policy and/or practice supported by the reported data?	☒	☐	☐	☐
11. Were the specific directives for new research appropriate?	☒	☐	☐	☐

Overall appraisal: Include ☒ Exclude ☐ Seek further info ☐

Comments (Including reason for exclusion)

____

____

____
